# Biogeographical Consequences of Cenozoic Tectonic Events within East Asian Margins: A Case Study of *Hynobius* Biogeography

**DOI:** 10.1371/journal.pone.0021506

**Published:** 2011-06-28

**Authors:** Jun Li, Cuizhang Fu, Guangchun Lei

**Affiliations:** 1 Ministry of Education Key Laboratory for Biodiversity Science and Ecological Engineering, and Institute of Biodiversity Science, Fudan University, Shanghai, China; 2 School of Nature Conservation, Beijing Forestry University, Beijing, China; University of Copenhagen, Denmark

## Abstract

Few studies have explored the role of Cenozoic tectonic evolution in shaping patterns and processes of extant animal distributions within East Asian margins. We select *Hynobius* salamanders (Amphibia: Hynobiidae) as a model to examine biogeographical consequences of Cenozoic tectonic events within East Asian margins. First, we use GenBank molecular data to reconstruct phylogenetic interrelationships of *Hynobius* by Bayesian and maximum likelihood analyses. Second, we estimate the divergence time using the Bayesian relaxed clock approach and infer dispersal/vicariance histories under the ‘dispersal–extinction–cladogenesis’ model. Finally, we test whether evolutionary history and biogeographical processes of *Hynobius* should coincide with the predictions of two major hypotheses (the ‘vicariance’/‘out of southwestern Japan’ hypothesis). The resulting phylogeny confirmed *Hynobius* as a monophyletic group, which could be divided into nine major clades associated with six geographical areas. Our results show that: (1) the most recent common ancestor of *Hynobius* was distributed in southwestern Japan and Hokkaido Island, (2) a sister taxon relationship between *Hynobius retardatus* and all remaining species was the results of a vicariance event between Hokkaido Island and southwestern Japan in the Middle Eocene, (3) ancestral *Hynobius* in southwestern Japan dispersed into the Taiwan Island, central China, ‘Korean Peninsula and northeastern China’ as well as northeastern Honshu during the Late Eocene–Late Miocene. Our findings suggest that Cenozoic tectonic evolution plays an important role in shaping disjunctive distributions of extant *Hynobius* within East Asian margins.

## Introduction

Understanding the role of tectonic evolution of earth plates in shaping biodiversity distribution patterns is one of the central aims in historical biogeography [1]. In East Asian margins, there were intensive rifting and extensional tectonics associated with block rotations and volcanism in the Cenozoic, owing to the interaction of the Eurasian, Pacific and Philippine Sea plates [2]–[7]. The most important tectonic events were the formation of islands (e.g., Japanese Islands, Taiwan Island) and the opening of a series of linked marginal seas (e.g. Japan Sea) in the period between the Eocene and Early Pliocene. These tectonic activities resulted in East Asian marginal fragmentation [4], [8], [9]. How the fragmentation processes of East Asian margins affected dispersal and vicariance histories of the region's biota is of particular interest [10]–[12]. Previously, the few biogeographical studies on East Asian margins that selected relatively young taxa as model organisms, only recovered one biogeographical pattern, which is the dispersal from East Asian continent to islands (e.g., Japanese Islands, Taiwan Island) during the Late Miocene–Pleistocene [10]–[12]. Biogeographical events within East Asian margins before the Late Miocene in the Cenozoic have remained unexplored.

**Figure 1:**
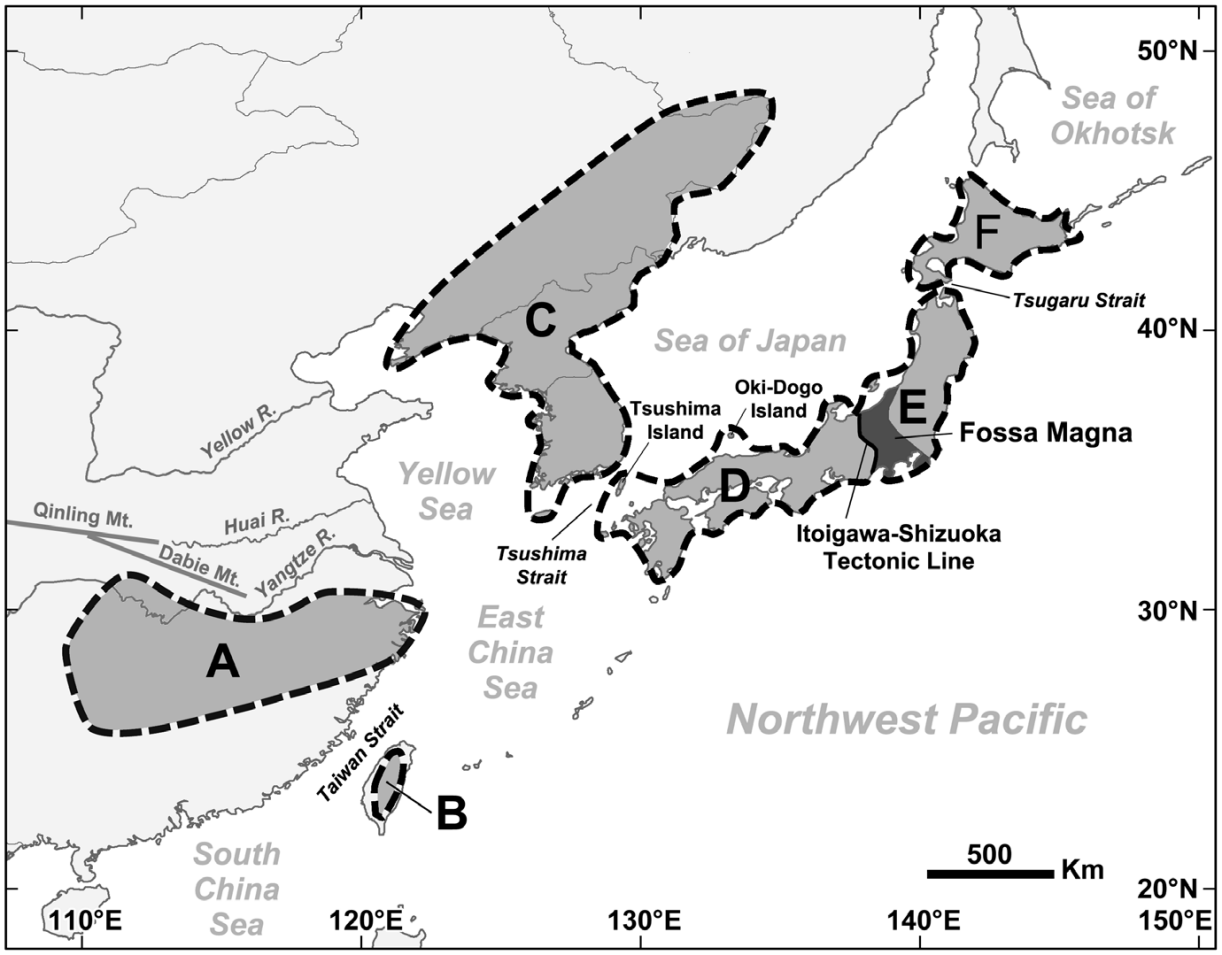
Geographical ranges of East Asian *Hynobius* salamanders. Contemporary distribution ranges of East Asian *Hynobius* are divided into six clearly-defined areas within East Asian margins: A, central China; B, Taiwan Island; C, Korean Peninsula and northeastern China; D, southwestern Japan; E, northeastern Honshu Japan; F, Hokkaido Island.

*Hynobius* is the most speciose genus in the family Hynobiidae, comprising one species *Hynobius turkestanicus* distributed in Central Asia and thirty-one species in East Asian margins [13] (see taxonomy in Table S1). It is a monophyletic taxon which originated in the early Cenozoic [14]. According to their contemporary distribution ranges, apart from *H. turkestanicus*, all other species are endemic to one (very rarely two) of the following six clearly-defined areas within the East Asian margins (Figure 1; Table S1): five species in central China, three species in the ‘Korean Peninsula and northeastern China’, five species in Taiwan Island, one species in Hokkaido Island, thirteen species in southwestern Japan, two species in northeastern Honshu, and two species in both southwestern Japan and northeastern Honshu [15]–[18].

**Figure 2:**
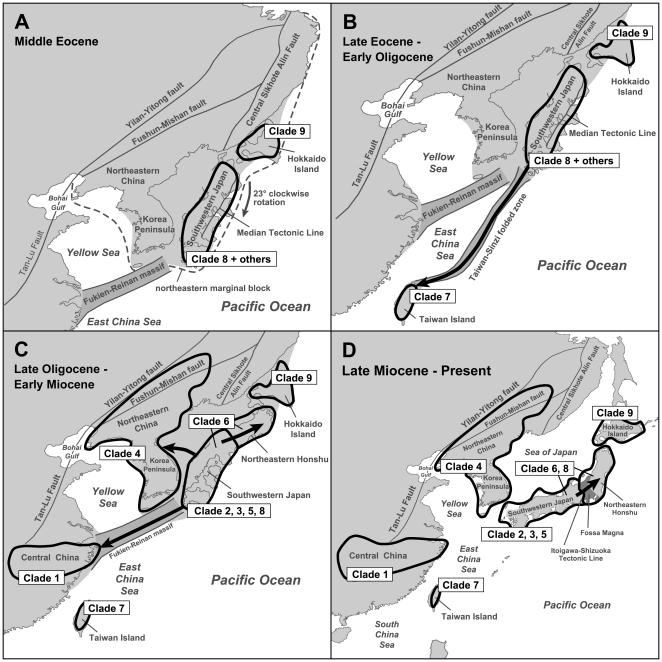
Sketch maps of main evolutionary scenarios of East Asian *Hynobius*. Biogeographical scenarios inferred in this study were put onto the paleo-maps, which were drawn from geological knowledge in previous studies [4]–[9], [19], [20], [24], [26]–[27]. (A) vicariance between Hokkaido Island and southwestern Japan in Middle Eocene; (B) dispersal from southwestern Japan to Taiwan Island followed by vicariance between the two regions in Late Eocene-Early Oligocene; (C) dispersal from southwestern Japan to the Korean Peninsula and northeastern China, to central China and to northeastern Honshu in Late Oligocene-Early Miocene; (D) dispersal from southwestern Japan to northeastern Honshu in Late Miocene-present. Clades are defined in Figure 3.

On the basis of a complete review of the literature on the Cenozoic tectonic history in East Asian margins, the following six independent geological events were identified to have had the potential to cause vicariance or dispersal, which may have shaped disjunctive distributions of extant *Hynobius* in East Asian margins. The six geological events are as follows: (1) Geological event 1: The first stage of rifting in East Asian margins formed a continuous block called as the ‘northeastern marginal block’ [5], [7], [19]. This block includes the ‘Korean Peninsula and northeastern China’, southwestern Japan, Sikhote Alin and Hokkaido Island (Figure 2A). This geological event resulted in the separation of ‘northeastern marginal block’ from all remaining East Asian margins in the Late Cretaceous–Early Tertiary [5], [7], [19]. (2) Geological event 2: Hokkaido Island was situated adjacent to Sikhote Alin, at around its present latitude, since the Late Cretaceous [19]. Hokkaido Island was separated from southwestern Japan as a result of the circa 23° clockwise rotation of southwestern Japan relative to the ‘northeastern marginal block’ in the Early Tertiary (Figure 2A) [7], [20]. (3) Geological event 3: A land bridge called as the ‘Fukien–Reinan Massif’ (Figure 2A–C) formed in the Late Mesozoic [21] and broke up in the late Early Miocene [22], [23], separating the Yellow Sea from East China Sea. The position of ‘Fukien–Reinan Massif’ remains controversial. A geological hypothesis suggests the ‘Fukien–Reinan Massif’ as the connection between the Korean Peninsula and central China [21], [24]. Another hypothesis suggests that the ‘Fukien–Reinan Massif’ linked central China and southwestern Japan [25], [26]. (4) Geological event 4: Southwestern Japan was seperated from the ‘Korean Peninsula and northeastern China’ with the opening of Japan Sea in the Middle Miocene [27], [28]. (5) Geological event 5: The formation of Taiwan Island. A popular geological hypothesis suggests that the formation of Taiwan Island was the results of ‘Penglai Orogeny’ in the Late Miocene, followed by the opening of Taiwan Strait in the Early Pliocene [29], [30]. Another hypothesis suggests that a part of the mountain ranges in present-day Taiwan Island is a relict area of a land bridge called as the ‘Taiwan–Sinzi Folded Zone’ (Figure 2B) connecting southwestern Japan with Taiwan Island [21], [24], [31], [32]. This land bridge formed in the Late Eocene–Early Oligocene [8] and broke up in the Middle Oligocene [33]. (6) Geological event 6: Northeastern Honshu originated from low latitudes and reached East Asian margins since the Early Oligocene [34], [35]. Northeastern Honshu was separated from southwestern Japan with the opening of a seaway near the ‘Fossa Magna’ (Figure 1) during the late Early Miocene–Late Miocene [9].

### Hypothesis 1: ‘vicariance’ hypothesis

On the basis of the geological evidence as mentioned above, we hypothesized ancestral distribution of the most recent common ancestor of *Hynobius* throughout the ‘northeastern marginal block’ and central China before the Late Cretaceous, followed by cladogenesis through vicariance owing to the sequential fragmentation of East Asian margins in the Cenozoic (hypothesis 1). Our hypothesis 1 could be further divided into six sub-hypotheses (1A–F) as follows: (1) Hypothesis 1A: We hypothesized that the most recent common ancestor of *Hynobius* were widespread across the ‘northeastern marginal block’ and central China before the formation of ‘northeastern marginal block’ in the Late Cretaceous–Early Tertiary (geological event 1). The hypothesis 1A predicted that the crown age of *Hynobius* should predate timing of the formation of ‘northeastern marginal block’. (2) Hypothesis 1B: We hypothesized that a sister group relationship between *Hynobius* in ‘central China–Taiwan Island’ and all remaining species from ‘Hokkaido Island–southwestern Japan–northeastern Honshu–Korean Peninsula and northeastern China’ as a result of a vicariance event between the ‘northeastern marginal block’ and all remaining areas within East Asian margins in the Late Cretaceous–Early Tertiary, driven by the first stage of East Asian marginal rifting (geological event 1). (3) Hypothesis 1C: We hypothesized that origin of the only species (*Hynobius retardatus*) in Hokkaido Island was the results of a vicariance event between Hokkaido Island and southwestern Japan in the Early Tertiary, driven by the clockwise rotation of southwestern Japan (geological event 2). (4) Hypothesis 1D: We hypothesized that a sister group relationship between *Hynobius* in southwestern Japan and those from the ‘Korean Peninsula and northeastern China’ was the results of a vicariance event between the two areas in the Middle Miocene, driven by the Japan Sea opening (geological event 4). (5) Hypothesis 1E: We hypothesized that a sister group relationship between *Hynobius* in Taiwan Island and those in central China was the results of vicariance between the two areas in the Early Pliocene, driven by the opening of Taiwan Strait (geological event 5). (6) Hypothesis 1F: We hypothesized that a sister group relationship between *Hynobius* in southwestern Japan and those in northeastern Honshu was the results of a vicariance event between the two areas in the late Early Miocene–Late Miocene, driven by the opening of a seaway near the ‘Fossa Magna’ (geological event 6). Each of the hypotheses 1B–F predicted that the divergence timing of cladogenesis should coincide with timing of the associated vicariance event as mentioned in each sub-hypothesis.

### Hypothesis 2: ‘out of southwestern Japan’ hypothesis

A region with the highest species richness for a specific taxon is often identified as an area of origin [1]. For *Hynobius* salamanders, southwestern Japan has the highest species richness (15 species in southwestern Japan and 1–5 species in other areas; Table S1). Thus, we hypothesized ancestral distribution of the most recent common ancestor of *Hynobius* in southwestern Japan, followed by cladogenesis through dispersal from there in different steps (hypothesis 2). Our hypothesis 2 could be further divided into six sub-hypotheses (2A–F) as follows: (1) Hypothesis 2A: We hypothesized ancestral distribution of the most recent common ancestor of *Hynobius* in southwestern Japan as a result of the formation of ‘northeastern marginal block’ in the Late Cretaceous–Early Tertiary (geological event 1). The hypothesis 2A predicted that the the crown age of *Hynobius* should coincide with or postdate timing of the formation of ‘northeastern marginal block’. (2) Hypothesis 2B: We hypothesized ancestral *Hynobius* in southwestern Japan dispersing via the ‘Fukien–Reinan Massif’ into central China before a vicariance event between the two areas in the late Early Miocene as a result of the break-up of ‘Fukien–Reinan Massif’ (geological event 3). The hypothesis 2B predicted that *Hynobius* in central China are nested within those from southwestern Japan and the divergence timing of cladogenesis should predate timing of the vicariance event. (3) Hypothesis 2C: We hypothesized that ancestral *Hynobius* in southwestern Japan dispersed via a land connection into Hokkaido Island before a vicariance event between the two areas in the Early Tertiary, driven by the clockwise rotation of southwestern Japan (geological event 2). The hypothesis 2C predicted that *Hynobius* in Hokkaido Island are nested within those from southwestern Japan and the divergence timing of cladogenesis should predate timing of the vicariance event. (4) Hypothesis 2D: We hypothesized that ancestral *Hynobius* in southwestern Japan dispersed via a land connection into the ‘Korean Peninsula and northeastern China’ before a vicariance event between the two areas in the Middle Miocene, driven by the opening of Japan Sea (geological event 4). The hypothesis 2D predicted that *Hynobius* in the ‘Korean Peninsula and northeastern China’ are nested within those from southwestern Japan and the divergence timing of cladogenesis should predate timing of the vicariance event. (5) Hypothesis 2E: We hypothesized that ancestral *Hynobius* in southwestern Japan dispersed via the ‘Taiwan–Sinzi Folded Zone’ into Taiwan Island before a vicariance event between the two areas in the Middle Oligocene, driven by the break-up of Taiwan–Sinzi Folded Zone (geological event 5). The hypothesis 2E predicted that *Hynobius* in Taiwan Island are nested within those from southwestern Japan and the divergence timing of cladogenesis should predate timing of the vicariance event. (6) Hypothesis 2F: We hypothesized that ancestral *Hynobius* in southwestern Japan dispersed into northeastern Honshu via a land connection before a vicariance event between the two areas in the late Early Miocene–Late Miocene, driven by the opening of a seaway near the ‘Fossa Magna’ (geological event 6). The hypothesis 2F predicted that *Hynobius* in northeastern Honshu are nested within those from southwestern Japan and the divergence timing of cladogenesis should predate timing of the vicariance event.

In the present study, we use GenBank molecular data and a fossil calibration to infer phylogenetic interrelationships of East Asian *Hynobius* and a chronogram. Then, biogeographical scenarios for dispersal or/and vicariance histories in East Asian margins are inferred on the basis of ancestral area reconstruction. Finally, we test whether evolutionary history and biogeographical processes of *Hynobius* should coincide with predictions of two major biogeographical hypotheses (the ‘vicariance’/‘out of southwestern Japan’ hypothesis). Our findings should provide insight into the biogeographical consequences of Cenozoic geological events within East Asian margins.

## Results

### Phylogenetic inference

**Figure 3:**
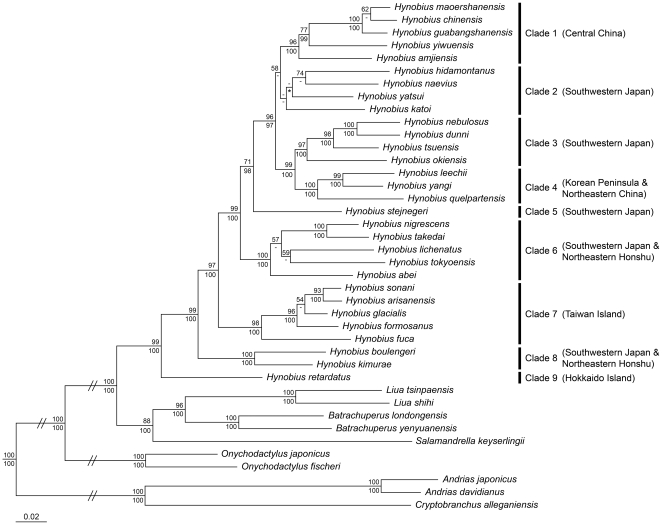
Topology of maximum likelihood analysis based on combined data of mitochondrial genes. The four outgroup taxa are not shown. Values above each node are bootstrap confidence (BS) results for maximum likelihood (ML) analysis and values below each node are Bayesian posterior probabilities (PP) for Bayesian (BA) analysis. BS values lower than 50% and PP values lower than 95% are indicated by ‘-’. Asterisks (*) indicate topological incongruence between ML and BA topology. ‘//’ indicates 1/4 of the branch length. Nine defined clades with associated distributional areas in bracket are listed on the right. Information on distributions of each species is provided in Table S1 and Figure 4.

Bayesian (BA) and Maximum likelihood (ML) analyses yielded similar topology of trees, and confirmed East Asian *Hynobius* as a monophyletic group. *Hynobius* could be divided into nine major clades (defined in Figure 3) with strong supports (Bayesian posterior probabilities, PP = 0.97–1.00; bootstrap values, BS = 71–100%) for Clades 1 and 3–7, and with weak supports for Clade 2 and a sister-group relationship between Clades 1 and 2. Using a reduced taxon sampling (excluding species with only one or three genes), further analyses recovered strong supports for Clade 2 (PP = 1.00; BS = 87%) and still weak supports for a sister-group relationship between Clades 1 and 2 (for details see Text S2 and Figure S2).

Major clades recovered in the *Hynobius* phylogeny show clear associations with six geographical areas (Figure 3). Species from Clades 2–3 and Clade 5 are only distributed in southwestern Japan. Species from Clade 6 and Clade 8 are distributed in southwestern Japan or/and northeastern Honshu. Species from Clade 1 are only distributed in central China, Clade 4 in the ‘Korean Peninsula and northeastern China’, Clade 7 in Taiwan Island and Clade 9 in Hokkaido Island.

### Divergence time estimation and ancestral area reconstruction

**Figure 4:**
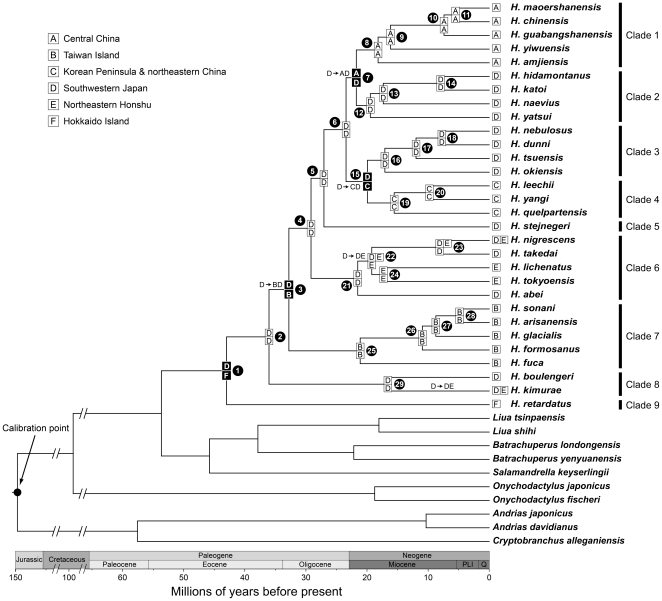
Chronogram and ancestral area reconstructions of East Asian *Hynobius*. The chronogram was estimated based on the maximum likelihood tree in BEAST. The ancestral area reconstruction was performed under the dispersal-extinction-caldogenesis (DEC) model in LAGRANGE. Area delimitations (A–F) are provided in Figure 1. Arrows on branches represent dispersal directions. Black cells with letters represent vicariant events. Black circles with numbers represent serial numbers of internal nodes. The four outgroup taxa are not shown. ‘Q’ and ‘PLI’ are abbreviations of the Quaternary and Pliocene. Clades are defined in Figure 3.

The chronogram is shown in Figure 4. The stem and crown age of *Hynobius* are circa 54 and 43 Ma, respectively. Timing of the internal nodes of *Hynobius* is provided in Table 1. The results of ancestral area reconstruction are shown in Figure 4 and Table 1. For each node, ancestral range subdivision/inheritance scenarios are shown on its descendant branch. Inferred scenarios for most of nodes were strongly supported (relative probability, RP = 0.78–1.00), whereas the scenarios for nodes 2, 6 and 21 had only moderate supports (RP = 0.56–0.62) (Table 1).

**Table 1:** Estimated ages and reconstructed ancestral areas for internal nodes within the phylogeny of East Asian *Hynobius*.

	Estimated age (Ma)		Ancestral Area	
Node	mean	95% CI	split	relative probability
1	42.99	33.99–52.92	D | F	0.78
2	36.04	28.43–44.04	D | D	0.56
3	32.79	25.94–40.04	D | B	0.99
4	29.17	23.20–35.64	D | D	0.95
5	27.05	21.36–33.18	D | D	0.96
6	23.42	18.66–28.81	D | D	0.62
7	21.74	17.15–26.78	A | D	1.00
8	18.18	14.09–22.75	A | A	1.00
9	16.15	12.18–20.45	A | A	1.00
10	7.39	4.86–10.03	A | A	1.00
11	5.49	3.43–7.79	A | A	1.00
12	19.45	14.70–24.39	D | D	1.00
13	17.34	12.99–21.98	D | D	1.00
14	7.96	0.00–16.85	D | D	1.00
15	19.93	15.58–24.57	D | C	1.00
16	17.08	12.91–21.44	D | D	1.00
17	11.97	8.58–15.60	D | D	1.00
18	7.75	5.05–10.59	D | D	1.00
19	15.56	11.71–19.73	C | C	1.00
20	9.80	6.54–13.09	C | C	1.00
21	21.51	16.19–27.28	D | D	0.58
22	19.21	14.46–24.34	DE | E	0.87
23	8.09	5.03–11.48	DE | D	0.91
24	17.31	12.44–22.15	E | E	1.00
25	21.11	14.37–28.17	B | B	1.00
26	10.94	7.17–15.06	B | B	1.00
27	8.74	5.53–12.30	B | B	1.00
28	4.86	2.55–7.49	B | B	1.00
29	16.59	10.75–23.24	D | D	0.79

Shown are mean values and 95% confidence interval (CI) of ages, and the subdivision/inheritance scenario (‘split’) with the highest relative probability (RP) based on a dispersal-extinction-cladogenesis model in LAGRANGE. Serial numbers of nodes are defined in Figure 4. Abbreviations of area delimitations (A–F) are provided in Figure 1.

Dispersal and vicariance events (indicated by arrows and black cells in Figure 4, separately) were inferred as follows (Figure 4, Table 1): (1) East Asian *Hynobius* originated in southwestern Japan (D) and Hokkaido Island (F); (2) a vicariance event occurred between southwestern Japan and Hokkaido Island on node 1; (3) a dispersal event occurred from southwestern Japan to Taiwan Island (B) between nodes 2 and 3, followed by a vicariance event between the two areas on node 3; (4) a dispersal event occurred from southwestern Japan to the ‘Korea Peninsula and northeastern China’ (C) between nodes 6 and 15, followed by a vicariance event between the two areas on node 15; (5) a dispersal event occurred from southwestern Japan to central China (A) between nodes 6 and 7, followed by a vicariance event between the two areas on node 7; (6) two dispersal events occurred from southwestern Japan to northeastern Honshu (E) between nodes 21 and 22 and between node 29 and its tip.

## Discussion

### Phylogeny and divergence time of *Hynobius*

Our study provides the first well-resolved phylogeny of East Asian *Hynobius* with a near-complete taxon sampling (30 of 32 species). One of our major sources of molecular data is from Larson et al. [36]. They sampled 24 *Hynobius* species and divided the genus into six clades using the maximum parsimony approach. However, they failed to resolve interrelationships among their clades, especially those corresponding to our Clades 1–6.

The stem age of *Hynobius* (circa 54 Ma) estimated in the present study is consistent with the results reported by recent studies using the same calibration or multiple fossil calibrations [14], [37], [38].

### Origin of extant *Hynobius*

Our timing of the crown age of extant *Hynobius* in the Early Tertiary (node 1 in Figure 4) contradicts with the prediction of hypothesis 1A and coincides with the prediction of hypothesis 2A. However, the origin of extant *Hynobius* in southwestern Japan and Hokkaido Island recovered by our ancestral area reconstruction is slightly different from the hypothesis 2A for ancestral distribution of the most recent common ancestor of *Hynobius* in southwestern Japan. An alternative hypothesis is that *Hynobius* probably originated in the ‘northeastern marginal block’ (see the geological event 1 in the section of introduction) including the ‘Korean Peninsula and northeastern China’, southwestern Japan, Sikhote Alin and Hokkaido Island (Figure 2A), followed by extinction in the ‘Korean Peninsula and northeastern China’ and Sikhote Alin owing to the nearly synchronous occurence of strong volcanism during the formation of the block [7]. Further studies are needed within the framework of Hynobiidae to explain the origin of extant *Hynobius*.

### Endemics in central China

*Hynobius* in central China (Clade 1) nested within those from southwestern Japan (Figure 3) recovered in the present study contradicts with the prediction of vicariance-based hypothesis 1B and coincides with the prediction of dispersal-based hypothesis 2B. Our timing of the divergence time between *Hynobius* sister groups from central China (Clade 1) and southwestern Japan (Clade 2) in the earlist Early Miocene (node 7 in Figure 4) predated the timing of a vicariance event between the two areas in the late Early Miocene as a result of the break-up of ‘Fukien–Reinan Massif’ (see the geological event 3 in the section of introduction) [22], [23], which is also consistent with the hypothesis 2B's prediction. The hypothesis 2B is further supported by our results of ancentral area reconstruction, which revealed a dispersal event of *Hynobius* from southwestern Japan into central China, followed by vicariance between the two areas (Figure 4).

If the inference is correct, our results support a geological hypothesis that the ‘Fukien–Reinan Massif’ linked central China and southwestern Japan [25], [26] and indirectly reject another hypothesis that the ‘Fukien–Reinan Massif’ extended from the Korean Peninsula to central China [21], [24]. Because the sister group relationship between Clade 1 and Clade 2 has weak statistical supports, this conclusion from our results should be taken with caution.

### Endemics in Hokkaido Island

The only species in Hokkaido Island, *Hynobius retardatus*, occupies a basal position in our *Hynobius* phylogeny, which contradicts with the dispersal-based hypothesis 2C. Our timing of the divergence time between *Hynobius retardatus* and all remaining species in the Early Tertiary (node 1 in Figure 4) overlapped with the timing of a vicariance event between Hokkaido Island and southwestern Japan in the Early Tertiary as a result of the clockwise rotation of southwestern Japan (see the geological event 2 in the section of introduction) [7], [20], which coincides with the prediction of vicariance-based hypothesis 1C. Vicariance between Hokkaido Island and southwestern Japan revealed by our ancentral area reconstruction further supports the hypothesis 1C.

### Endemics in the ‘Korean Peninsula and northeastern China’

*Hynobius* in the ‘Korean Peninsula and northeastern China’ nested within those from southwestern Japan recovered in the present study (Figure 3) contradicts with the prediction of vicariance-based hypothesis 1D and coincides with the prediction of dispersal-based hypothesis 2D. Our timing of the divergence time between *Hynobius* sister groups from southwestern Japan (Clade 3) and the ‘Korean Peninsula and northeastern China’ (Clade 4) in the Early Miocence (node 15 in Figure 4) predated the timing of a vicariance event between the two areas as a result of the Japan Sea opening in the Middle Miocene [27], [28], which is also consistent with the hypothesis 2D's prediction. We futher support the hypothesis 2D based on our results of ancentral area reconstruction, which suggests a dispersal event of *Hynobius* from southwestern Japan into the ‘Korean Peninsula and northeastern China’, followed by a vicariance event between the two areas (Figure 4).

### Endemics in Taiwan Island

*Hynobius* in Taiwan Island nested within those from southwestern Japan recovered in the present study (Figure 3) contradicts with the prediction of vicariance-based hypothesis 1E and coincides with the prediction of dispersal-based hypothesis 2E. Our timing of the stem age of Taiwan *Hynobius* in the Early Oligocene (node 3 in Figure 4) predated the timing of a vicariance event between southwestern Japan and Taiwan Island in the Middle Oligocene as a result of the break-up of ‘Taiwan–Sinzi Folded Zone’ (see the geological event 5 in the section of introduction) [33], which is also consistent with the hypothesis 2E's prediction. We futher support the hypothesis 2E based on our results of ancentral area reconstruction, which suggest a dispersal event from southwestern Japan into Taiwan Island, followed by vicariance between the two areas (Figure 4).

Our timing of the crown age of Taiwan *Hynobius* in the late Early Miocene (node 25 in Figure 4) supports a geological hypothesis that part of the mountain ranges in Taiwan Island is a relict area of the ‘Taiwan–Sinzi Folded Zone’ [21], [24], [31], [32] and rejects alternative hypothesis that entire areas of Taiwan Island originated in the Late Miocene–Early Pliocene [29], [30].

### *Hynobius* in northeastern Honshu

Our phylogeny show that *Hynobius* in northeastern Honshu nested among those from southwestern Japan belong to two distant taxon groups (Clades 6 and 9 in Figure 3). The origin of *Hynobius* in northeastern Honshu could be attributed to two dispersal events from southwestern Japan to northeastern Honshu (Figure 4). Our timing of the first dispersal event (between nodes 21 and 22; Figure 4) in the earlist Early Miocene predated the timing of a vicariance event between the two areas in the late Early Miocene–Late Miocene as a result of the opening of a seaway (see the geological event 6 in the section of introduction) [9], which contradicts with the prediction of vicariance-based hypothesis 1F and coincides with the prediction of dispersal-based hypothesis 2F. The second dispersal event (between node 29 and its tip; Figure 4) occurred after the Middle Miocene, which is not predicted by our hypotheses. An alternative hypothesis is that the second dispersal event from southwestern Japan to northeastern Honshu was due to the closure of the seaway reconnecting the two area after Late Miocene [9].

### Some caveats

We caution that in the present study, we assumed that probable biases with the ancestral area reconstruction approach and lack of *Hynobius* fossil evidence would not affect the inference of biogeographical scenarios. The ancestral area reconstruction approach is a ‘pattern before process’ approach, as argued by Crisp et al. [39]: ‘The logical problem with this type of approach is that a finite set of observations can be consistent with an almost unlimited set of alternative explanations’. In the present study, probable biases could not be avoided using the ancestral area reconstruction approach. Therefore, we explore biogeographical processes of *Hynobius* to make conclusions based on a process-based hypothesis-testing approach. The fossil record have been indicated to be crucially important for reliable divergence time estimates [39], [40]. Lacking *Hynobius* fossil evidence might result in biased divergence time estimates in the present study.

### A summary

Our results support most of the predictions of ‘out of southwestern Japan’ hypothesis as mentioned above. Biogeographical scenarios of *Hynobius* could be summarized as follows (Figure 2): (1) ancestral distribution of the most recent common ancestor of *Hynobius* in southwestern Japan and Hokkaido Island; (2) a sister taxon relationship between *Hynobius retardatus* and all remaining species was the results of a vicariance event between southwestern Japan and Hokkaido Island in the Early Tertiary, driven by the clockwise rotation of southwestern Japan (Figure 2A); (3) ancestral *Hynobius* in southwestern Japan dispersed via the ‘Taiwan-Sinzi Folded Zone’ into Taiwan Island in the Late Eocene–Early Oligocene (Figure 2B); (4) ancestral *Hynobius* in southwestern Japan dispersed via land connections into the ‘Korean Peninsula and northeastern China’ in the Late Oligocene–Early Miocene (Figure 2C); (5) ancestral *Hynobius* in southwestern Japan dispersed via the ‘Fukien–Reinan Massif’ into central China in the Late Oligocene–Early Miocene (Figure 2C); (6) ancestral *Hynobius* in southwestern Japan dispersed via land connections into northeastern Honshu twice in the period either the Early Miocene or after the Late Miocene (Figure 2C–D).

Similar biogeographical processes might be found in other taxa that possess distribution ranges in East Asian margins and evolutionary histories during the Cenozoic. Organisms used in previously biogeographical studies [10]–[12] are too young (Late Miocene–Pleistocene) to recover the biogeographical events within East Asian margins suggested by our study. The ‘out of southwestern Japan’ hypothesis could be an alternative explanation for disjunctive distributions of other old taxa in East Asian margins, but further studies are needed to test this hypothesis.

## Materials and Methods

### Taxa selection and molecular data

Among a total of thirty-two *Hynobius* species (Table S1), mitochondrial DNA sequences are available for thirty species from GenBank, including two protein-coding genes [Cytochrome *b* (*Cyt b*) and NADH dehydrogenase subunit 2 (*ND2*)], two ribosomal RNA (rRNA) genes (*12S rRNA* and *16S rRNA*) and six transfer RNA (tRNA) genes (*tRNA-Val*, *tRNA-Trp*, *tRNA-Ala*, *tRNA-Asn*, *tRNA-Cys* and *tRNA-Tyr*). Sequences from different individuals of a certain species were combined with caution (for details see Text S1). *Hynobius hirosei* and *H. turkestanicus* lack molecular data and were excluded from this study. Fourteen species were selected as outgroups for phylogenetic inference based on the results obtained by recent molecular studies of salamanders [14], [37]. These outgroups include seven close relatives of *Hynobius* in Hynobiidae, three species of Cryptobranchidae, and four species representing four other families (Rhyacotritonidae, Ambystomatidae, Salamandridae and Plethodontidae) respectively. Among all the 44 selected taxa, 36 taxa (circa 82%) possessed nine or all ten genes, while the remaining eight taxa possessed one or three genes. Details for a checklist of family and species, GenBank accession numbers and associated references are provided in Table S2. Details of the origin of molecular data sources for *Hynobius* species are described in supporting information (Text S1, Figure S1 and Table S4, S5, S6).

### Phylogenetic inference

Sequences of protein-coding genes (*Cyt b* and *ND2*) were aligned using CLUSTAL X ver. 1.83 [41]. The secondary structures of non-coding genes (*12S rRNA*, *16S rRNA* and six *tRNA*s) were estimated using RNAstructure ver. 5.2 [42], and were aligned using MUSCLE ver. 3.6 [43]. Ambiguous alignments were removed under Gblocks ver. 0.91b [44] using the ‘with half’ option and default block parameters. Partitioned Bayesian and maximum likelihood analyses were performed to reconstruct phylogenetic relationships of *Hynobius* based on the concatenated dataset of ten genes (44 taxa; total sequence length 4563 bp). A 12-partition scheme was applied: (1) the loop and stem regions of *12S rRNA* and *16S rRNA* were treated as separate partitions; (2) since the loop/stem regions of *tRNAs* were short (∼30–40 bp), all the loop regions of the six *tRNAs* were concatenated together as one partition, so were the stem regions; (3) the protein-coding genes were partitioned according to the codon positions, thus six partitions were applied for the two protein-coding genes.

For partitioned Bayesian (BA) analysis, each partition had an independent model of substitution suggested by jModeltest ver. 0.1.1 [45] using the Akaike Information Criterion (AIC). Selected models are provided in Table S3. Markov chains Monte Carlo (MCMC) were run for 10 million generations implemented in MrBayes ver. 3.1.2 [46]. Trees were sampled every 1000 generations. Stationarity was checked graphically by plotting log-likelihood scores in Tracer ver. 1.4.1 [47]. The first one million generations before stationarity were discarded as burnin and the remaining trees were used to build a consensus tree.

Partitioned maximum likelihood (ML) analysis was implemented using a rapid-hill-climbing algorithm in RAxML ver. 7.0.4 [48]. First, the best-scoring ML tree was inferred with 100 replications under the GTRMIX model. Then, a nonparametric bootstrap analysis of 1000 replications was conducted under the GTRCAT model to evaluate node robustness of the ML tree.

### Divergence time estimates

The estimation of divergence time was performed in BEAST ver. 1.6.1 [49]. We set the parameters of BEAST following the suggestions of Zhang & Wake [37]. The phylogenetic tree from the ML inference was used as a starting tree. We assumed a relaxed uncorrelated lognormal clock for rate variation model and a pure birth model (Yule process) for the tree prior. An independent substitution model was assigned to each partition according to the results of jModeltest ver. 0.1.1 [45]. The split between Hynobiidae and Cryptobranchidae was calibrated using a lognormal prior, allowing ‘hard’ minimum and ‘soft’ maximum constraints. The minimum age of the calibration point was decided using the earliest known cryptobranchoid fossil record *Chunerpeton tianyiense* [50], whose age was revised to be 140 Ma in Marjanović & Laurin [51]. The ‘soft’ maximum constraint was set to 170 Ma, referring to the origin of living salamanders [51]. A test run of six million generations was performed to optimize the scale factors of the priori function. The final Markov chain Monte Carlo (MCMC) was run for 50 million generation with a sampling frequency of 1000. Tracer ver. 1.4.1 [47] was used to check the stationarity, and the first five million generations were subsequently discarded as burnin.

### Area delimitation and biogeographical reconstruction

We compiled distribution data of *Hynobius* species from the published literatures listed in Table S1. Contemporary distribution ranges of *Hynobius* in East Asian margins were divided into six areas (see Figure 1) based on their disjunctive distribution patterns and possible biogeographical barriers. Each *Hynobius* species was then assigned to its associated area according to its contemporary distribution range. The six areas are: A, central China; B, Taiwan Island; C, the Korean Peninsula and northeastern China; D, southwestern Japan including the southwestern Honshu, Shikoku, Kyushu and adjacent small islands (e.g., the Tsushima and Oki-Dogo Islands); E, northeastern Honshu; F, Hokkaido Island. The three areas (A, B and C) were defined based on the disjunctive distributions of *Hynobius* in theses areas, and were named following Zhao [15]. The division of Japanese Islands into D, E and F was based on the Tsugaru Strait and Fossa Magna (see Figure 1) as biogeographical barriers. Fossa Magna (the Itoigawa-Shizuoka Tectonic Line) is a major transverse zone of Miocene tectonic depression [52], and has been indicated as a biogeographical barrier for the Japanese freshwater fish fauna [53], [54] and a *Hynobius* salamander, *H. katoi* [55]. Tsugaru Strait, separating Honshu Island from Hokkaido Island, was suggested as a biogeographical barrier of amphibians because no urodeles and only one anuran (*Hyla japonica*) were shared between Honshu and Hokkaido [56].

Ancestral areas of *Hynobius* were reconstructed under the dispersal-extinction-cladogenesis (DEC) model in LAGRANGE ver. 2.0.1 [57], [58]. The DEC model specifies instantaneous transition rates between discrete distribution ranges along phylogenetic branches, and uses the rates to access the range inheritance scenarios at cladogenesis events [58]. The analysis was conducted based on the coded distribution range as defined above, and the chronogram of *Hynobius* species estimated in BEAST ver. 1.6.1. The maximum number of ancestral areas was constrained to two, assuming that the dispersal ability of ancestors is similar to that of their extant descendants [59].

## Supporting Information

Figure S1 **Bayesian inference of the phylogeny of East Asian** ***Hynobius*** **.** Different specimens of the four species (*Hynobius lichenatus*, *H. retardatus*, *H. tokyoensis* and *H. yiwuensis*) were treated as different analytic units (shown with bold fonts). The four outgroup taxa are not shown. Bayesian posterior probabilities (PP) were given above each node (PP<95% not shown). ‘//’ indicates half of the branch length.
(TIF)

Figure S2 **Maximum likelihood inference for relationships among Clade 1–5 defined in** **Figure 3** **using reduced taxa.** Species possessed only one or three genes were excluded. The outgroup taxa (Clade 6) are not shown. Values above each node are bootstrap confidence (BS) results for maximum likelihood (ML) analysis and values below each node are Bayesian posterior probabilities (PP) for Bayesian (BA) analysis. BS values lower than 50% and PP values lower than 95% are indicated by ‘-’.
(TIF)

Table S1 **List of East Asian** ***Hynobius*** **salamanders with presence-absence distributional data in defined areas.**
(DOC)

Table S2 **List of East Asian** ***Hynobius*** **salamanders and outgroup species with GenBank accession numbers of ten mitochondrial genes.**
(DOC)

Table S3 **Substitution models selected in jModeltest using the Akaike Information Criterion (AIC).**
(DOC)

Table S4 **List of the species possessing sequences from a single specimen without taxonomic revision.**
(DOC)

Table S5 **List of the species possessing sequences from a single specimen, for which taxonomic revision is necessary.**
(DOC)

Table S6 **Uncorrected** ***p*** **-distance between NC_008084 and the** ***Cyt b*** **fragments from identified specimens of the five** ***Hynobius*** **species in the Taiwan Island.**
(DOC)

Text S1 **Origin of molecular data sources for** ***Hynobius*** **species.**
(DOC)

Text S2 **Re-examination of relationships among** ***Hynobius*** **Clades 1–5 defined in** **Figure 3** **using reduced taxon dataset.**
(DOC)
