# Supplementary figures and images for: Biogeographical Consequences of Cenozoic Tectonic Events within East Asian Margins: A Case Study of Hynobius Biogeography
Source: PLoS One. 2011 Jun 28;6(6):e21506. doi: 10.1371/journal.pone.0021506 (PMC3125272; doi:10.1371/journal.pone.0021506)

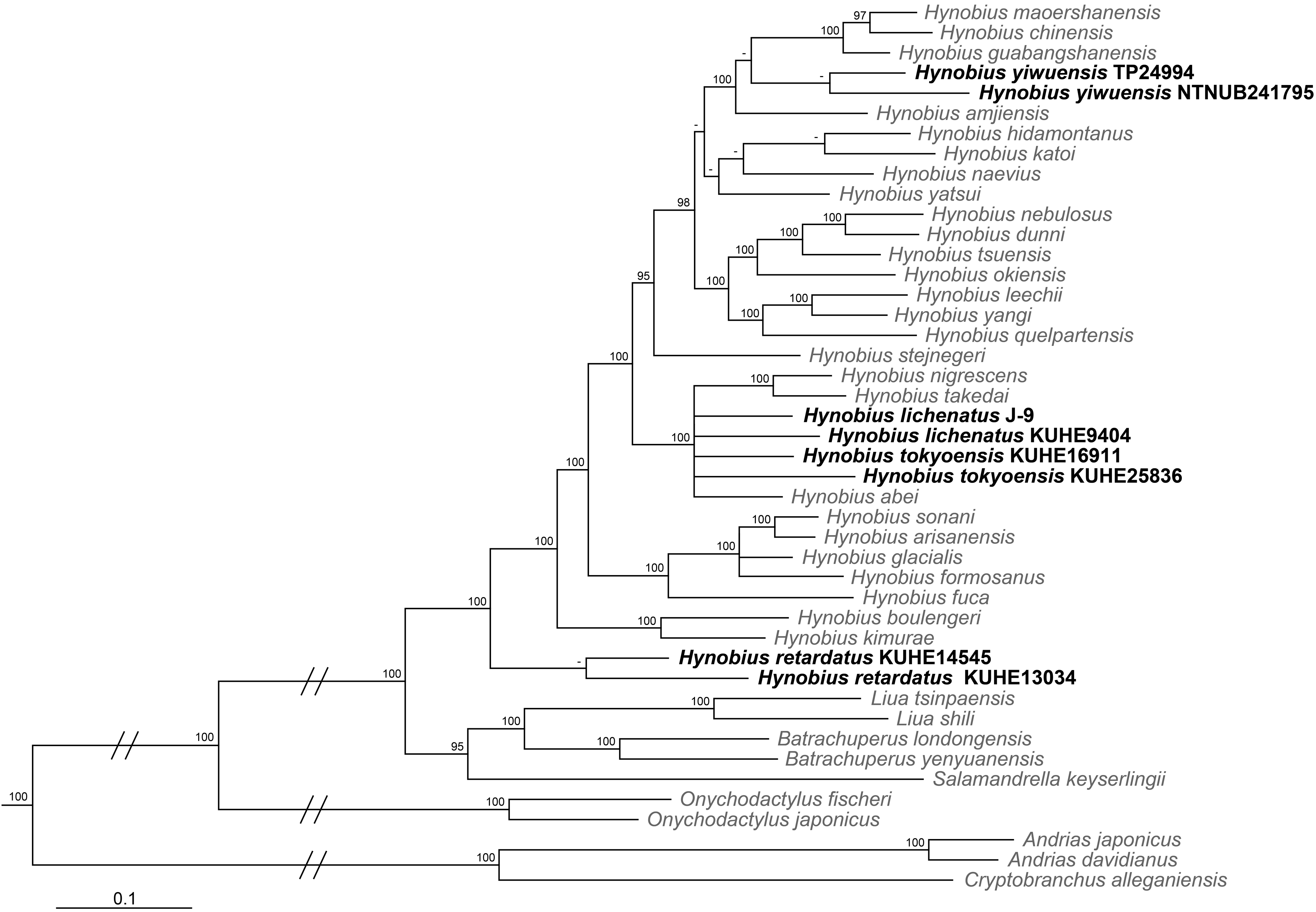

Supplement: Figure S1 — Bayesian inference of the phylogeny of East Asian Hynobius . Different specimens of the four species (Hynobius lichenatus, H. retardatus, H. tokyoensis and H. yiwuensis) were treated as different analytic units (shown with bold fonts). The four outgroup taxa are not shown. Bayesian posterior probabilities (PP) were given above each node (PP<95% not shown). ‘//’ indicates half of the branch length. (TIF) [file pone.0021506.s001.tif]

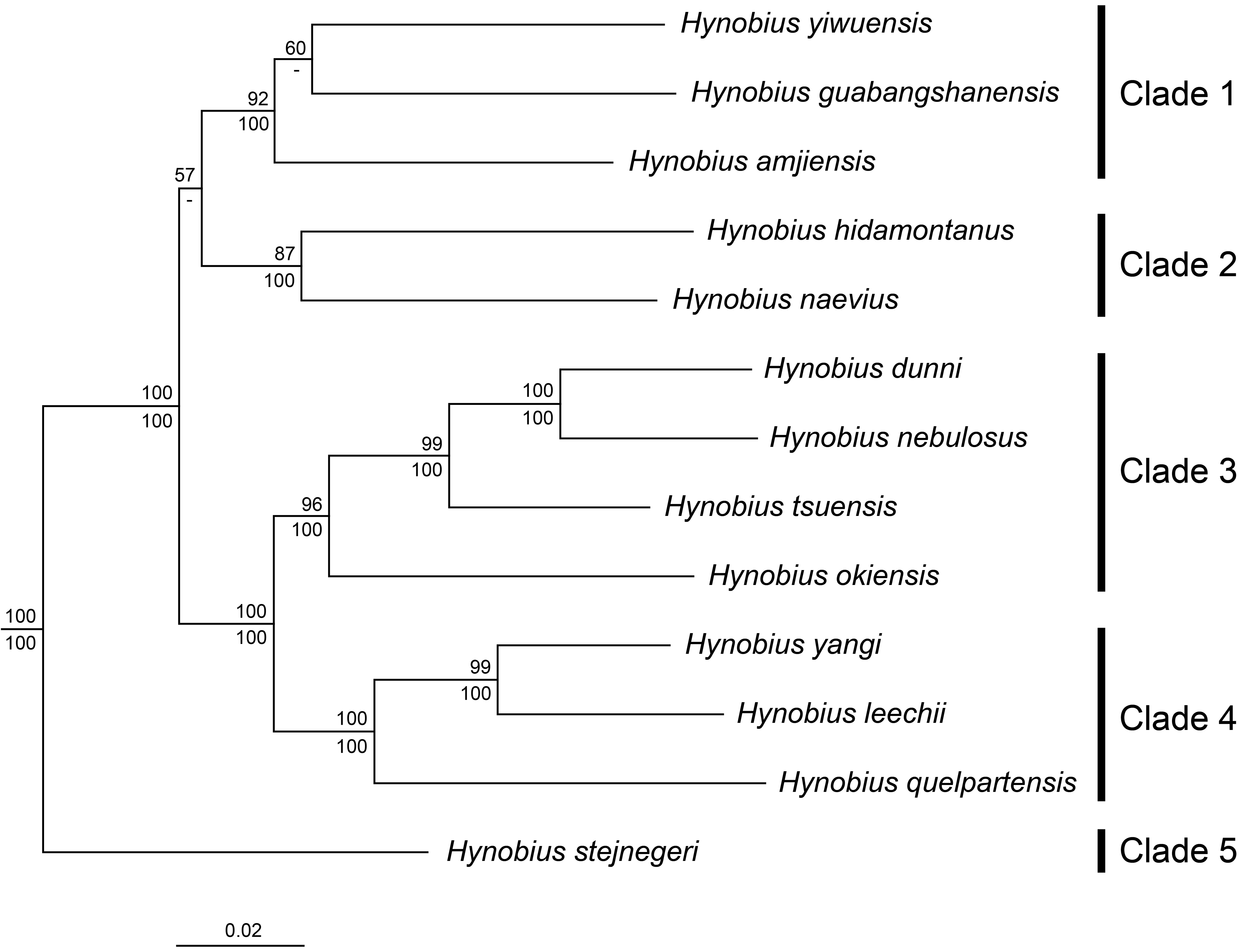

Supplement: Figure S2 — Maximum likelihood inference for relationships among Clade 1–5 defined in Figure 3 using reduced taxa. Species possessed only one or three genes were excluded. The outgroup taxa (Clade 6) are not shown. Values above each node are bootstrap confidence (BS) results for maximum likelihood (ML) analysis and values below each node are Bayesian posterior probabilities (PP) for Bayesian (BA) analysis. BS values lower than 50% and PP values lower than 95% are indicated by ‘-’. (TIF) [file pone.0021506.s002.tif]
