# Supplementary material for: Biogeographical Consequences of Cenozoic Tectonic Events within East Asian Margins: A Case Study of Hynobius Biogeography
Source: PLoS One. 2011 Jun 28;6(6):e21506. doi: 10.1371/journal.pone.0021506 (PMC3125272; doi:10.1371/journal.pone.0021506)
Supplement: Table S1 — List of East Asian Hynobius salamanders with presence-absence distributional data in defined areas. (DOC) [file pone.0021506.s003.doc]

**Table S1.** List of *Hynobius* salamander species with presence-absence distributional data in defined areas.

| Species | HI | SJ | NHJ | CC | TI | KPNC | CA |
| --- | --- | --- | --- | --- | --- | --- | --- |
| *Hynobius abei* Sato, 1934 |  | + |  |  |  |  |  |
| *Hynobius amjiensis* Gu, 1992 |  |  |  | + |  |  |  |
| *Hynobius arisanensis* Maki, 1922 |  |  |  |  | + |  |  |
| *Hynobius boulengeri* (Thompson, 1912) |  | + |  |  |  |  |  |
| *Hynobius chinensis* Günther, 1889 |  |  |  | + |  |  |  |
| *Hynobius dunni* Tago, 1931 |  | + |  |  |  |  |  |
| *Hynobius formosanus* Maki, 1922 |  |  |  |  | + |  |  |
| *Hynobius fuca* Lai and Lue, 2008 |  |  |  |  | + |  |  |
| *Hynobius glacialis* Lai and Lue, 2008 |  |  |  |  | + |  |  |
| *Hynobius guabangshanensis* Shen, Deng, and Wang, 2004 |  |  |  | + |  |  |  |
| *Hynobius hidamontanus* Matsui, 1987 |  | + |  |  |  |  |  |
| *Hynobius hirosei* Lantz, 1931 |  | + |  |  |  |  |  |
| *Hynobius katoi* Matsui, Kokuryo, Misawa, and Nishikawa, 2004 |  | + |  |  |  |  |  |
| *Hynobius kimurae* Dunn, 1923 |  | + | + |  |  |  |  |
| *Hynobius leechii* Boulenger, 1887 |  |  |  |  |  | + |  |
| *Hynobius lichenatus* Boulenger, 1883 |  |  | + |  |  |  |  |
| *Hynobius maoershanensis* Zhou, Jiang, and Jiang, 2006 |  |  |  | + |  |  |  |
| *Hynobius naevius* (Temminck and Schlegel, 1838) |  | + |  |  |  |  |  |
| *Hynobius nebulosus* (Temminck and Schlegel, 1838) |  | + |  |  |  |  |  |
| *Hynobius nigrescens* Stejneger, 1907 |  | + | + |  |  |  |  |
| *Hynobius okiensis* Sato, 1940 |  | + |  |  |  |  |  |
| *Hynobius quelpaertensis* Mori, 1928 |  |  |  |  |  | + |  |
| *Hynobius retardatus* Dunn, 1923 | + |  |  |  |  |  |  |
| *Hynobius sonani* (Maki, 1922) |  |  |  |  | + |  |  |
| *Hynobius stejnegeri* Dunn, 1923 |  | + |  |  |  |  |  |
| *Hynobius takedai* Matsui and Miyazaki, 1984 |  | + |  |  |  |  |  |
| *Hynobius tokyoensis* Tago, 1931 |  |  | + |  |  |  |  |
| *Hynobius tsuensis* Abé, 1922 |  | + |  |  |  |  |  |
| *Hynobius turkestanicus* Nikolskii, 1910 |  |  |  |  |  |  | + |
| *Hynobius yangi* Kim, Min, and Matsui, 2003 |  |  |  |  |  | + |  |
| *Hynobius yatsui* Oyama, 1947 |  | + |  |  |  |  |  |
| *Hynobius yiwuensis* Cai, 1985 |  |  |  | + |  |  |  |

HI, the Hokkaido Island; SJ, the southwestern Japan; NHJ, the northeastern Honshu, Japan; CC, the central China; TI, the Taiwan Island; KPNC, the Korean Peninsula and northeastern China; and CA, the Central Asia. The distributional information of each species was obtained from the literatures listed below the table.

**References**

AmphibiaWeb (2009) AmphibiaWeb: Information on amphibian biology and conservation. [web application]. Available at http://amphibiaweb.org/. Accessed at April, 2009. Berkeley: AmphibiaWeb.

Chen Q, Qing LY, Zeng XM (2008) Review of systematic study on *Hynobius*. Sichuan J Zool 27: 468-477.

Fei L, Hu SQ, Ye CY, Huang YZ (2006) Fauna Sinica. Amphibia Vol. 1: General accounts of Amphibia; Gymnophiona and Urodela. Beijing: Science Press.

Fei L, Ye CY, Jiang JP, Xie F, Huang YZ (2005) An illustrated key to Chinese amphibians. Chengdu: Sichuan Publishing House of Science and Technology.

Frost DR (2009) Amphibian Species of the World: an Online Reference. Version 5.3 (12 February, 2009). Electronic Database accessible at http://research.amnh.org/herpetology/amphibia/. New York: American Museum of Natural History.

Fu JZ, Hayes M, Liu ZJ, Zeng XM (2003) Genetic divergence of the southeastern Chinese salamanders of the genus *Hynobius*. Acta Zool Sinica 49: 585-591.

Guo KJ, Mi XQ, Deng XJ (2008) Breeding ecology of *Hynobius guabangshanensis*. Chin J Ecol 27: 77-82.

Hangui J, Wakahara M, Michimae H (2009) Plastic responses to different types of cue: Predator-induced and deep-water-induced polyphenisms in a salamander *Hynobius retardatus*. Zool Sci 26: 119-124.

Hasumi M, Iwasawa H (1990) Seasonal changes in body shape and mass in the salamander, *Hynobius nigrescens*. J Herpetol 24: 113-118.

Hasumi M, Iwasawa H (1993) Geographic variation in the pes of the salamander *Hynobius lichenatus*: A comparison with tetradactyl *Hynobius hidamontanus* and pentadactyl *Hynobius nigrescens*. Zool Sci 10: 1017-1027.

Iizuka K, Kezer J, Seto T (1989) Karyotypes of two rare species of hynobiid salamanders from Taiwan, *Hynobius sonani* (Maki) and *Hynobius formosanus* Maki (Urodela). Genetica 78: 105-110.

Ikebe C, Kohno S (1979) Cytogenetic studies of Hynobiidae (Urodela). I. Karyotypes of *Hynobius nebulosus nebulosus* (Schlegel) and *Hynobius nebulosus tokyoensis* Tago. Proc Jpn Acad Ser B 55: 436-440.

Ikebe C, Kuro-o M, Yamada H, Kohno S (1990) Cytogenetic studies of Hynobiidae (Urodela). X. Morphological variation of chromosome 10 in ten pond-type *Hynobius* from Korea and Japan, with comments on phylogenetic relationships. J Evol Biol 3: 155-170.

Ikebe C, Gu HQ, Ruan RW, Kohno S (1998) Chromosomes of *Hynobius chinensis* Günther and *Hynobius amjiensis* Gu from China, and comparison with those of 19 other *Hynobius* species. Zool Sci 15: 981-987.

Ikebe C, Kuro-o M, Ohtani H, Kawase Y, Matsui T, et al. (2005) Cytogenetic studies of Hynobiidae (Urodela). XIX. Morphological variation of sex chromosomes pairing behavior of sex lampbrush chromosomes in *Hynobius quelpaertensis* (Mori) from Cheju Island, South Korea. Chromosome Res 13: 157-167.

Iwasaki F, Wakahara M (1999) Adaptable larval life histories in different populations of the salamander, *Hynobius retardatus*, living in various habitats. Zool Sci 16: 667-674.

Izumisawa Y, Ikebe C, Kuro-o M, Kohno S (1984) Cytogenetic studies of Hynobiidae (Urodela). IX. Karyological characteristics of *Hynobius abei* Sato by means of R- and C- banding. Experientia 46: 104-106.

Jiang H, Chen KJ, She SM, Liu JB, Lin S, et al. (2005) Histology observation of sex gland and morphology of a possibly new variety of hynobiid species – *Hynobius huayuanensis* sp. nov. J Hunan Agric Univ (Nat Sci) 31: 183-186.

Kim JB, Min MS, Matsui M (2003) A new species of lentic breeding Korean salamander of the genus *Hynobius* (Amphibia: Urodela). Zool Sci 20: 1163-1169.

Kim JB, Matsui M, Nishikawa K (2007) Genetic relationships among salamanders of the genus *Hynobius* (Amphibia, Caudata) from Korea and Southwestern Japan. Zool Sci 24: 1128-1133.

Kuramoto M (1972) Low natural fertilization rate in *Hynobius tsuensis* Abe (Amphibia: Urodela). Herpetologica 28: 38-41.

Kuro-o M, Ikebe C, Izumisawa Y, Fujinuki Y, Sasaki K, et al. (2002) Cytogenetic studies of Hynobiidae (Urodela). XVIII. A ZZ/ZW sex determining mechanism in a hynobiid salamander species, *Hynobius tokyoensis* Tago. Cytogenet Genome Res 99: 194-199.

Kusano T, Inoue M (2008) Long-term trends toward earlier breeding of Japanese amphibians. J Herpetol 42: 608-614.

Kusano T, Miyashita K (1984) Dispersal of the salamander, *Hynobius nebulosus tokyoensis*. J Herpetol 18: 349-353.

Kusano T, Ueda T, Nakagawa H (2006) Body size and age structure of breeding populations of the salamander, *Hynobius tokyoensis* (Caudata: Hynobiidae). Curr Herpetol 25: 71-78.

Lai JS, Lue KY (2007) Distribution and population dynamics of Alishan salamander (*Hynobius arisanensis*) in Alishan areas. BioFormosa 42: 105-117.

Lai JS, Lue KY (2008) Two new *Hynobius* (Caudata: Hynobiidae) salamanders from Taiwan. Herpetologica 64: 63-80.

Lee HY, Kim YR, Yang DE, Yang SY (1998) The genetic differentiation of the mitochondrial cytochrome *b* gene of Korean salamanders. Korean J Genet 20: 155-162.

Lee JH, Park D (2008) Morphological characteristics of the lateral line receptors of *Hynobius leechii* (Urodela: Hynobiidae). Zool Sci 25: 642-652.

Matsui M (1987) Isozyme variation in salamanders of the *nebulosus-lichenatus* complex of the genus *Hynobius* from Eastern Honshu, Japan, with a description of a new species. Jpn J Herpetol 12: 50-64.

Matsui M (2007) The Chugoku Mountains viewed from amphibian fauna. Taxa 22: 34-37.

Matsui M, Miyazaki K (1984) *Hynobius takedai* (Amphibia, Urodela), a new species of salamander from Japan. Zool Sci 1: 665-671.

Matsui M, Seto T, Miyazaki K (1985) The karyotype of *Hynobius takedai* M. Matsui et Miyazaki, 1984, with comments on the karyotypic relationships among Japanese salamanders of the genus *Hynobius*. Jpn J Genet 60: 119-123.

Matsui M, Misawa Y, Nishikawa K (2009) Morphological variation in a Japanese salamander, *Hynobius kimurae* (Amphibia, Caudata). Zool Sci 26: 87-95.

Matsui M, Sato T, Tanabe S, Hayashi T (1992) Electrophoretic analyses of systematic relationships and status of two hynobiid salamanders from Hokkaido (Amphibia: Caudata). Herpetologica, 48: 408-416.

Matsui M, Misawa Y, Nishikawa K, Tanabe S (2000) Allozymic variation of *Hynobius kimurae* Dunn (Amphibia, Caudata). Comp Biochem Physiol B 125: 115-125.

Matsui M, Nishikawa K, Tanabe S, Misawa Y (2001) Systematic status of *Hynobius tokyoensis* (Amphibia: Urodela) from Aichi Prefecture, Japan: a biochemical survey. Comp Biochem Physiol B 130: 181-189.

Matsui M, Kokuryo Y, Misawa Y, Nishikawa K (2004) A new species of salamander of the genus *Hynobius* from Central Honshu, Japan. Zool Sci 21: 661-669.

Matsui M, Nishikawa K, Utsunomiya T, Tanabe S (2006) Geographic allozyme variation in the Japanese clouded salamander, *Hynobius nebulosus* (Amphibia: Urodela). Biol J Linn Soc Lond, 89: 311-330.

Matsui M, Nishikawa K, Misawa Y, Tanabe S (2007) Systematic relationships of *Hynobius okiensis* among Japanese salamanders (Amphibia: Caudata). Zool Sci 24: 746-751.

Matsui M, Iwasawa H, Takahashi H, Hayashi T, Kumakura M (1992) Invalid specific status of *Hynobius sadoensis* Sato: Electrophoretic evidence (Amphibia: Caudata). J Herpetol 26: 308-315.

Matsui M, Tominaga A, Hayashi T, Misawa Y, Tanabe S (2007) Phylogenetic relationships and phylogeography of *Hynobius tokyoensis* (Amphibia: Caudata) using complete sequences of cytochrome *b* and control region genes of mitochondrial DNA. Mol Phylogenet Evol 44: 204-216.

Michimae H (2006) Differentiated phenotypic plasticity in larvae of the cannibalistic salamander *Hynobius retardatus*. Behav Ecol Sociobiol 60: 205-211.

Michimae H (2007) Differentiated egg size of the cannibalistic salamander *Hynobius retardatus*. J Ethol 25: 153-158.

Misawa Y, Matsui M (1997) Larval life history variation in two populations of the Japanese salamander *Hynobius kimurae* (Amphibia, Urodela). Zool Sci 14: 257-262.

Misawa Y, Matsui M (1999) Age determination by skeletochronology of the Japanese salamander *Hynobius kimurae* (Amphibia, Urodela). Zool Sci 16: 845-851.

Nishikawa K, Matsui M (2008) A comparative study on the larval life history in two populations of *Hynobius boulengeri* from Kyushu, Japan (Amphibia: Urodela). Curr Herpetol 27: 9-22.

Nishikawa K, Matsui M, Tanabe S (2005) Biochemical phylogenetics and historical biogeography of *Hynobius boulengeri* and *H. stejnegeri* (Amphibia: Caudata) from the Kyushu region, Japan. Herpetologica 61: 54-62.

Nishikawa K, Sato S, Matsui M (2008) A note on the clutch size and shape of egg sacs of *Hynobius boulengeri* from the Sobo-Katamuki Mountains, Kyushu, Japan (Urodela: Hynobiidae). Curr Herpetol 27: 29-34.

Nishikawa K, Matsui M, Tanabe S, Sato S (2001) Geographic enzyme variation in a Japanese salamander, *Hynobius boulengeri* Thompson (Amphibia: Caudata). Herpetologica 57: 281-294.

Nishikawa K, Matsui M, Kokuryo Y, Misawa Y (2005) Karyotype of a Japanese salamander *Hynobius katoi* and its implication on breeding ecology (Amphibia: Caudata). Zool Sci 22: 805-807.

Nishikawa K, Matsui M, Tanabe S, Sato S (2007) Morphological and allozymic variation in *Hynobius boulengeri* and *H. stejnegeri* (Amphibia: Urodela: Hynobiidae). Zool Sci 24: 752-766.

Oh DJ, Chang MH, Oh HS, Jung YH (2007) The complete mitochondrial DNA sequence of the Jeju salamander, *Hynobius quelpaertensis*, and the phylogenetic relationships among the Hynobiidae. Korean J Genet 29: 331-341.

Okada Y (1934) A contribution toward a check list of the urodeles of Japan. Copeia 1934: 16-19.

Oyama J (1930) *Hynobius tagoi* Dunn not a valid species. Copeia 1930: 155-156.

Park D, Park SR (2000) Multiple insemination and reproductive biology of *Hynobius leechii*. J Herpetol 34: 594-598.

Sakamoto M, Nishikawa K, Matsui M (2005) Two types of *Hynobius naevius* from the central region of Kyushu Island, Japan (Caudata: Hynobiidae). Curr Herpetol 24: 67-77.

Sakamoto M, Tominaga A, Matsui M, Sakata K, Uchino A (2009) Phylogeography of *Hynobius yatsui* (Amphibia: Caudata) in Kyushu, Japan. Zool Sci 26: 35-47.

Seto T, Matsui M (1984) Karyotype of the Japanese salamander, *Hynobius abei*. Experientia 40: 874.

Shannon FA (1956) The reptiles and amphibians of Korea. Herpetologica 12: 22-49.

Shen YH, Deng XJ, Wang B (2004) A new hynobiid species *Hynobius guabangshanensis* from Hunan Province, China (Amphibia: Hynobiidae). Acta Zool Sinica 50: 209-215.

Su HJ (2008) New distribution records of the *Hynobius* species in Guizhou province. J Mountain Agric Biol 27: 170-172.

Sun PX, Jiang AQ, Guo SS, Zhang GQ, Jiang TM (2005) *Hynobius guabangshanensis*, biological characteristic, resource quantity and discussion on protective measures. Chin J Fish 18: 70-73.

Takahara T, Miyasaka H, Genkaikato M, Kohmatsu Y (2008) Length-weight relationships in six amphibian species of Japan. Curr Herpetol 27: 43-45.

Takahashi H (1990) Egg size variation among various breeding habitats in the salamander *Hynobius lichenatus*. Ecol Res 5: 393-398.

Tominaga A, Matsui M (2007) Estimation of the type locality of *Hynobius naevius* (Temminck and Schlegel, 1838), a salamander from Japan (Amphibia: Caudata). Zool Sci 24: 940-944.

Tominaga A, Matsui M (2008) Taxonomic status of a salamander species allied to *Hynobius naevius* and a reevaluation of *Hynobius naevius yatsui* Oyama, 1947 (Amphibia, Caudata). Zool Sci 25: 107-114.

Tominaga A, Matsui M, Nishikawa K, Sato S (2003) Occurrence of two types of *Hynobius naevius* in Northern Kyushu, Japan (Amphibia: Urodela). Zool Sci 20: 1467-1476.

Tominaga A, Matsui M, Nishikawa K, Tanabe S (2006) Phylogenetic relationships of *Hynobius naevius* (Amphibia: Caudata) as revealed by mitochondrial 12S and 16S rRNA genes. Mol Phylogenet Evol 38: 677-684.

Tominaga A, Matsui M, Nishikawa K, Tanabe S, Sato S (2005) Genetic differentiations of *Hynobius naevius* (Amphibia: Hynobiidae) as revealed by allozyme analysis. Biochem Syst Ecol 33: 921-937.

Tominaga A, Matsui M, Nishikawa K, Tanabe S, Sato S (2005) Morphological discrimination of two genetic groups of a Japanese salamander, *Hynobius naevius* (Amphibia, Caudata). Zool Sci 22: 1229-1244.

Wakahara M (1995) Cannibalism and the resulting dimorphism in larvae of a salamander *Hynobius retardatus*, inhabited in Hokkaido, Japan. Zool Sci 12: 467-473.

Wang SP, Chen R, Huang ZY, Yang SQ, Gu HQ, et al. (1998) Taxonomic and evolutionary studies using RAPD on Zhejiang tailed amphibians. J Fudan Univ (Nat Sci) 37: 485-490.

Wang X, Wu M, Zhang Y, Wang WJ, Liu MY, et al. (2007) On the re-discovery of *Hynobius chinensis* Günther, 1989 from type-locality and its description after 116 years. Sichuan J Zool 26: 57-58.

Wang YH, Wang XL, Fang SG, Wu M (2005) DNA fingerprinting analysis of some species of Hynobiidae genera and a discuss of their classification. J Zhejiang Univ (Sci Ed) 32: 79-82.

Xiong JL (2008) The comparative anatomy of skeleton of the Chinese Hynobiidae salamanders and description of a new genus. Dissertation for Doctoral Degree. Chengdu: Sichuan University.

Xiong JL, Qing LY, Zeng XM, Zhao EM (2008) Karyotype of *Hynobius guabangshanensis* (Urodela: Hynobiidae). Sichuan J Zool 27: 236-238.

Xiong JL, Chen Q, Zeng XM, Zhao EM, Qing LY (2007) Karyotypic, morphological, and molecular evidence for *Hynobius yunanicus* as a synonym of *Pachyhynobius shangchengensis* (Urodela: Hynobiidae). J Herpetol 41: 664-671.

Yang L, Gong DJ, Mu M (2008) Present research situation and resource conservation of Hynobiidae (Amphibia: Urodela). Chin J Ecol 27: 111-116.

Yang SY, Kim JB, Min MS, Suh JH, Suk HY (1997) Genetic and phenetic differentiation among three forms of Korean salamander *Hynobius leechii*. Korean J Biol Sci 1: 247-257.

Zeng XM, Fu JZ (2004) Low genetic diversity in Chinese *Hynobius leechii*, with comments on the validity of *Hynobius mantchuricus*. Amphib-Reptil 25: 119-122.

Zeng XM, Fei L, Ye CY, Jiang JP (1997) The karyotypes of 3 species in genus *Hynobius* and *Sallamandrella* *keyserlingii*. Zool Res 18: 341-345.

Zhao EM (1990) Notes on some taxonomic problems in Chinese salamanders with a revised list. In: Zhao EM, editor. From Water onto Land. Beijing: China Forestry Press. pp. 217-220.

Zhao EM, Chang HW, Zhao H, Adler K (1990) Revised checklist of Chinese Amphibia and Reptilia. Sichuan J Zool 19: 196-207.

Zhou F, Jiang AW, Jiang DB (2006) A new species of the genus *Hynobius* from Guangxi Zhuang Autonomous Region, China (Caudata, Hynobiidae). Acta Zootaxonomica Sinica 31: 670-674.
