# Supplementary material for: Biogeographical Consequences of Cenozoic Tectonic Events within East Asian Margins: A Case Study of Hynobius Biogeography
Source: PLoS One. 2011 Jun 28;6(6):e21506. doi: 10.1371/journal.pone.0021506 (PMC3125272; doi:10.1371/journal.pone.0021506)
Supplement: Table S2 — List of East Asian Hynobius salamanders and outgroup species with GenBank accession numbers of ten mitochondrial genes. (DOC) [file pone.0021506.s004.doc]

**Table S2.** List of *Hynobius* salamander and out-group species with GenBank accession numbers of ten mitochondrial genes.

| **Speices** | **Specimen** | **Location** | **Reference** | **Cytb** | **12S** | **16S** | **ND2** | **tRNA-Val** | **tRNAs (Trp, Ala, Asn, Cys, Tyr)** |
| --- | --- | --- | --- | --- | --- | --- | --- | --- | --- |
| Hynobiidae |  |  |  |  |  |  |  |  |  |
| *Hynobius abei* | KUHE13514 | - | a |  | AY915987 | AY915987 | AY915939 | AY915987 | AY915939 |
| *Hynobius amjiensis* | - | Longwang Mountain, Zhejiang, China | b | NC_008076 | NC_008076 | NC_008076 | NC_008076 | NC_008076 | NC_008076 |
| **Hynobius arisanensis* | - | Nantou, Taiwan, China | b | NC_008084 | NC_008084 | NC_008084 | NC_008084 | NC_008084 | NC_008084 |
| *Hynobius boulengeri* | KUHE25653 | Kamikitayama-mura, Nara, Japan | c, d | AB266675 | AB201671 | AB201706 |  |  |  |
|  | KUHE25655 | Kamikitayama-mura, Nara, Japan | a |  | AY915994 | AY915994 | AY915946 | AY915994 | AY915946 |
| *Hynobius chinensis* | - | Yichang, Hubei, China | e | EF076244 |  |  |  |  |  |
| *Hynobius dunni* | KUHE24848 | - | a |  | AY915974 | AY915974 | AY915926 | AY915974 | AY915926 |
| *Hynobius formosanus* | NTNUB201695 | Taichung, Taiwan, China | f | DQ652201 |  |  |  |  |  |
| *Hynobius fuca* | NTNUB201746 | Taoyuan, Taiwan, China | f | DQ652194 |  |  |  |  |  |
| *Hynobius glacialis* | NTNUB201676 | Taichung, Taiwan, China | f | DQ652203 |  |  |  |  |  |
| **Hynobius guabangshanensis* | - | Qiyang, Hunan, China | b | NC_008088 | NC_008088 | NC_008088 | NC_008088 | NC_008088 | NC_008088 |
| *Hynobius hidamontanus* | KUHE9484 | - | a |  | AY915983 | AY915983 | AY915935 | AY915983 | AY915935 |
| *Hynobius katoi* | KUHE37128 | Misakubo-cho, Shizuoka, Japan | c | AB266673 |  |  |  |  |  |
| *Hynobius kimurae* | KUHE22370 | - | b |  | AY915995 | AY915995 | AY915947 | AY915995 | AY915947 |
|  | KUHE-UN | Kyoto-shi, Kyoto, Japan | d |  | AB201670 | AB201705 |  |  |  |
|  | KUHE16689 | Otsu-shi, Shiga, Japan | c | AB266674 |  |  |  |  |  |
| *Hynobius leechii* | - | Changbai Mountain, Jilin, China | b | NC_008079 | NC_008079 | NC_008079 | NC_008079 | NC_008079 | NC_008079 |
| *Hynobius lichenatus* | J-9 | - | a |  | AY915988 | AY915988 | AY915940 | AY915988 | AY915940 |
|  | KUHE9404 | Omagari-shi, Akita, Japan | c | AB266670 |  |  |  |  |  |
| *Hynobius maoershanensis* | CIB70079 | Xingan, Guangxi, China | g | AB548372 |  |  |  |  |  |
| *Hynobius naevius* | KUHE28584 | Tara-cho, Saga, Japan | c, d | AB266672 | AB201659 | AB201694 |  |  |  |
|  | KUHE12984 | Kitakyushu-shi, Fukuoka, Japan | a |  | AY915985 | AY915985 | AY915937 | AY915985 | AY915937 |
| *Hynobius nebulosus* | KUHE24693 | Isahaya-shi, Nagasaki, Japan | c, d | AB445786 | AB201668 | AB201703 |  |  |  |
|  | KUHE24698 | Isahaya-shi, Nagasaki, Japan | a |  | AY915973 | AY915973 | AY915925 | AY915973 | AY915925 |
| *Hynobius nigrescens* | KUHE17924 | Kami-machi, Miyagi, Japan | a, g | AB548378 | AY915991 | AY915991 | AY915943 | AY915991 | AY915943 |
| *Hynobius okiensis* | KUHE18917 | Dogo Island, Shimane, Japan | a |  | AY915979 | AY915979 | AY915931 | AY915979 | AY915931 |
| *Hynobius quelpartensis* | - | Ara-dong, Jeju Island, South Korea | i | NC_010224 | NC_010224 | NC_010224 | NC_010224 | NC_010224 | NC_010224 |
| *Hynobius retardatus* | KUHE14545 | - | a |  | AY915996 | AY915996 | AY915948 | AY915996 | AY915948 |
|  | KUHE13034 | Ebetsu, Hokkaido, Japan | j | AB363609 |  |  |  |  |  |
| *Hynobius sonani* | NTNUB201726 | Nantou, Taiwan, China | f | DQ652205 |  |  |  |  |  |
| *Hynobius stejnegeri* | KUHE14955 | Gokase-cho, Miyazaki, Japan | a |  | AY915986 | AY915986 | AY915938 | AY915986 | AY915938 |
| *Hynobius takedai* | KUHE24764 | - | a |  | AY915990 | AY915990 | AY915942 | AY915990 | AY915942 |
| *Hynobius tokyoensis* | KUHE16911 | - | a |  | AY915989 | AY915989 | AY915941 | AY915989 | AY915941 |
|  | KUHE25836 | Hachioji-shi, Tokyo, Japan | c | AB266640 |  |  |  |  |  |
| *Hynobius tsuensis* | KUHE18367 | - | a |  | AY915975 | AY915975 | AY915927 | AY915975 | AY915927 |
| **Hynobius yangi* | HLc10 | South Korea ('Form C') | a |  | AY915977 | AY915977 | AY915929 | AY915977 | AY915929 |
|  | NTNUB201703 | Pusan, South Korea | f | DQ652231 |  |  |  |  |  |
| **Hynobius yatsui* | KUHE24969 | Saeki-shi, Oita, Japan | d |  | AB201663 | AB201698 |  |  |  |
|  | - | Saeki-shi, Oita, Japan | k | AB297522 |  |  |  |  |  |
| **Hynobius yiwuensis* | TP24994 | Zhejiang, China | a |  | AY915982 | AY915982 | AY915934 | AY915982 | AY915934 |
|  | NTNUB241795 | Xiaoshan, Zhejiang, China | f | DQ652229 |  |  |  |  |  |
| *Batrachuperus londongensis* | - | Emei Mountain, Sichuan, China | b | NC_008077 | NC_008077 | NC_008077 | NC_008077 | NC_008077 | NC_008077 |
| *Batrachuperus yenyuanensis* | - | Luoji Mountain, Sichuan, China | b | DQ333818 | DQ333818 | DQ333818 | DQ333818 | DQ333818 | DQ333818 |
| *Liua shihi* | - | Badong, Hubei, China | b | NC_008078 | NC_008078 | NC_008078 | NC_008078 | NC_008078 | NC_008078 |
| *Liua tsinpaensis* | - | Zhouzhi, Shaanxi, China | b | NC_008081 | NC_008081 | NC_008081 | NC_008081 | NC_008081 | NC_008081 |
| *Onychodactylus fischeri* | - | Changbai Mountain, Jilin, China | b | NC_008089 | NC_008089 | NC_008089 | NC_008089 | NC_008089 | NC_008089 |
| *Onychodactylus japonicus* | J-5 | Japan | a |  | AY915971 | AY915971 | AY915923 | AY915971 | AY915923 |
|  | J52 | Hirakawa-shi, Aomori, Japan | l | AB452812 |  |  |  |  |  |
| *Salamandrella keyserlingii* | - | Xiao Xing'an Ling, Heilongjiang, China | b | NC_008082 | NC_008082 | NC_008082 | NC_008082 | NC_008082 | NC_008082 |
| Cryptobranchidae |  |  |  |  |  |  |  |  |  |
| *Andrias davidianus* | - | Longsheng, Guangxi, China | m | AJ492192 | AJ492192 | AJ492192 | AJ492192 | AJ492192 | AJ492192 |
| *Andrias japonicus* | - | - | n | AB208679 | AB208679 | AB208679 | AB208679 | AB208679 | AB208679 |
| *Cryptobranchus alleganiensis* | - | United States | a, h, o, p | AB445785 | AY915968 | DQ283263 | AY916039 | AY915968 | AY916039 |
| Rhyacotritonidae |  |  |  |  |  |  |  |  |  |
| *Rhyacotriton variegatus* | MVZ222581 | Mendocino, California, United States | q | NC_006331 | NC_006331 | NC_006331 | NC_006331 | NC_006331 | NC_006331 |
| Ambystomatidae |  |  |  |  |  |  |  |  |  |
| *Ambystoma mexicanum* | - | - | r | NC_005797 | NC_005797 | NC_005797 | NC_005797 | NC_005797 | NC_005797 |
| Salamandridae |  |  |  |  |  |  |  |  |  |
| *Salamandrina terdigitata* | MVZ178848 | Stazzemese, Lucca, Italy | s | EU880332 | EU880332 | EU880332 | EU880332 | EU880332 | EU880332 |
| Plethodontidae |  |  |  |  |  |  |  |  |  |
| *Plethodon petraeus* | MVZ222650 | Walker, Georgia, United States | q | NC_006334 | NC_006334 | NC_006334 | NC_006334 | NC_006334 | NC_006334 |

The reference for each sequence is marked by letters (a-s). The asterisks represent that the scientific names for the sequences are revised according to recent taxonomy studies. For some species, sequences were derived from different sources. These sequences were concatenated together in the phylogenetic analyses (Text S1).

**Reference**

(a) Macey JR, Weisrock DW, Fang Z, Matsui M, Larson A, et al. (submitted to Genbank in 2005) Molecular phylogenetics of the Hynobiidae (Amphibia: Caudata): evidence for an old northern Asian fauna. [Note: Their molecular data have been used in Larson et al. (2003). (Larson A, Weisrock DW, Kozak KH (2003) Phylogenetic systematics of salamanders (Amphibia: Urodela), a review. In: Server DM, editor. Reproductive biology and phylogeny of Urodela (Amphibia). Enfield: NH Science Publishers. pp. 31-108.)]

(b) Zhang P, Chen YQ, Zhou H, Liu YF, Wang XL, et al. (2006) Phylogeny, evolution, and biogeography of Asiatic salamanders (Hynobiidae). Proc Natl Acad Sci U S A 103: 7360-7365.

(c) Matsui M, Tominaga A, Hayashi T, Misawa Y, Tanabe S (2007) Phylogenetic relationships and phylogeography of *Hynobius tokyoensis* (Amphibia: Caudata) using complete sequences of cytochrome *b* and control region genes of mitochondrial DNA. Mol Phylogenet Evol 44: 204-216.

(d) Tominaga A, Matsui M, Nishikawa K, Tanabe S (2006) Phylogenetic relationships of *Hynobius naevius* (Amphibia: Caudata) as revealed by mitochondrial 12S and 16S rRNA genes. Mol Phylogenet Evol 38: 677-684.

(e) Zhang Y, Wu M, Wang WJ, Wang X (unpublished) Direct submission to Genbank. (Note: EF076244 from a specimen of the type location for *Hynobius chinensis*. Personal communications with one of authors, Prof. Wu M.)

(f) Lai JS, Lue KY (2008) Two new *Hynobius* (Caudata: Hynobiidae) salamanders from Taiwan. Herpetologica 64: 63-80.

(g) Nishikawa K, Jiang JP, Matsui M, Mo YM, Chen XH, et al. (2010) Invalidity of *Hynobius yunanicus* and molecular phylogeny of *Hynobius* salamander from continental China (Urodela, Hynobiidae). Zootaxa 2426: 65-67.

(h) Matsui M, Tominaga A, Liu WZ, Tanaka-Ueno T (2008) Reduced genetic variation in the Japanese giant salamander, *Andrias japonicas* (Amphibia: Caudata). Mol Phylogenet Evol 49: 318-326.

(i) Oh DJ, Chang MH, Oh HS, Jung YH (2007) The complete mitochondrial DNA sequence of the Jeju salamander, *Hynobius quelpaertensis*, and the phylogenetic relationships among the Hynobiidae. Korean J Genet 29: 331-341.

(j) Matsui M, Yoshikawa N, Tominaga A, Sato T, Takenaka S, et al. (2008) Phylogenetic relationships of two *Salamandrella* species as revealed by mitochondrial DNA and allozyme variation (Amphibia: Caudata: Hynobiidae). Mol Phylogenet Evol 48: 84-93.

(k) Sakamoto M, Tominaga A, Matsui M (2009) Phylogeography of *Hynobius yatsui* (Amphibia: Caudata) in Kyushu, Japan. Zool Sci 26: 35-47.

(l) Yoshikawa N, Matsui M, Nishikawa K, Kim JB, Kryukov A (2008) Phylogenetic relationships and biogeography of the Japanese clawed salamander, *Onychodactylus japonicas* (Amphibia: Caudata: Hynobiidae), and its congener inferred from the mitochondrial cytochrome *b* gene. Mol Phylogenet Evol 49: 249-259.

(m) Zhang P, Chen YQ, Liu YF, Zhou H, Qu LH (2003) The complete mitochondrial genome of the Chinese giant salamander, *Andrias davidianus* (Amphibia: Caudata). Gene 311: 93-98.

(n) Okamoto K, Suizu Y, Mizuno K, Tanigawa S, Sako K, et al. (unpublished) The giant salamander - comparison of mitochondrial whole genome between the Japanese and Chinese species.

(o) Frost DR, Grant T, Faivovich J, Bain RH, Haas A, et al. (2006) The amphibian tree of life. Bull Am Mus Nat Hist 297: 1-291.

(p) Weisrock DW, Harmon LJ, Larson A (2005) Resolving deep phylogenetic relationships in salamanders: analyses of mitochondrial and nuclear genomic data. Syst Biol 54: 758-777.

(q) Mueller RL, Macey JR, Jaekel M, Wake DB, Boore JL (2004) Morphological homoplasy, life history evolution, and historical biogeography of plethodontid salamanders inferred from complete mitochondrial genomes. Proc Natl Acad Sci U S A 101: 13820-13825.

(r) Arnason U, Gullberg A, Janke A, Joss J, Elmerot C (2004) Mitogenomic analyses of deep gnathostome divergence: a fish is a fish. Gene 333: 61-70.

(s) Zhang P, Papenfuss TJ, Wake MH, Qu LH, Wake DB (2008) Phylogeny and biogeography of the family Salamandridea (Amphibia: Caudata) inferred from complete mitochondrial genomes. Mol Phylogenet Evol 49: 586-597.
