# Supplementary material for: Biogeographical Consequences of Cenozoic Tectonic Events within East Asian Margins: A Case Study of Hynobius Biogeography
Source: PLoS One. 2011 Jun 28;6(6):e21506. doi: 10.1371/journal.pone.0021506 (PMC3125272; doi:10.1371/journal.pone.0021506)
Supplement: Table S3 — Substitution models selected in jModeltest using the Akaike Information Criterion (AIC). (DOC) [file pone.0021506.s005.doc]

**Table S3.** Substitution models selected in jModeltest using the Akaike Information Criterion (AIC).

| No. of partition | Partition | Model |
| --- | --- | --- |
| 1 | *Cyt b*, codon 1 | GTR+I+Γ |
| 2 | *Cyt b*, codon 2 | HKY+Γ |
| 3 | *Cyt b*, codon 3 | GTR+Γ |
| 4 | *ND2*, codon 1 | GTR+I+Γ |
| 5 | *ND2*, codon 2 | GTR+Γ |
| 6 | *ND2*, codon 3 | GTR+I+Γ |
| 7 | *12S rRNA*, loop region | HKY+I+Γ |
| 8 | *12S rRNA*, stem region | SYM+I+Γ |
| 9 | *16S rRNA*, loop region | GTR+Γ |
| 10 | *16S rRNA*, stem region | GTR+I+Γ |
| 11 | *tRNAs*, loop region | GTR+I+Γ |
| 12 | *tRNAs*, stem region | HKY+I+Γ |

*Cyt b*, Cytochrome *b* gene; *ND2*, NADH dehydrogenase subunit 2 gene; two ribosomal RNA (rRNA) genes: *12S rRNA* and *16S rRNA*; and six transfer RNA (*tRNAs*) genes: *tRNA-Val*, *tRNA-Trp*, *tRNA-Ala*, *tRNA-Asn*, *tRNA-Cys* and *tRNA-Tyr*.
