# Supplementary material for: Biogeographical Consequences of Cenozoic Tectonic Events within East Asian Margins: A Case Study of Hynobius Biogeography
Source: PLoS One. 2011 Jun 28;6(6):e21506. doi: 10.1371/journal.pone.0021506 (PMC3125272; doi:10.1371/journal.pone.0021506)
Supplement: Table S4 — List of the species possessing sequences from a single specimen without taxonomic revision. (DOC) [file pone.0021506.s006.doc]

**Table S4.** List of the species possessing sequences from a single specimen without taxonomic revision.

| Scientic Name | Voucher No. | Genbank accession No. | Reference |
| --- | --- | --- | --- |
| *Hynobius abei* | KUHE13514 | AY915939 & AY915987 | [1] |
| *Hynobius dunni* | KUHE24848 | AY915926 & AY915974 | [1] |
| *Hynobius hidamontanus* | KUHE9484 | AY915935 & AY915983 | [1] |
| *Hynobius okiensis* | KUHE18917 | AY915931 & AY915979 | [1] |
| *Hynobius stejnegeri* | KUHE14955 | AY915938 & AY915986 | [1] |
| *Hynobius takedai* | KUHE24764 | AY915942 & AY915990 | [1] |
| *Hynobius tsuensis* | KUHE18367 | AY915927 & AY915975 | [1] |
| *Hynobius amjiensis* | Nodata | Genome NC_008076 | [2] |
| *Hynobius leechii* | Nodata | Genome NC_008079 | [2] |
| *Hynobius quelpartensis* | Nodata | Genome NC_010224 | [11] |
| *Hynobius nigrescens* | KUHE17924 | AY915943, AY915991 & AB548378 | [1], [12] |

The references were given in Text S1.
