# Supplementary material for: Biogeographical Consequences of Cenozoic Tectonic Events within East Asian Margins: A Case Study of Hynobius Biogeography
Source: PLoS One. 2011 Jun 28;6(6):e21506. doi: 10.1371/journal.pone.0021506 (PMC3125272; doi:10.1371/journal.pone.0021506)
Supplement: Table S5 — List of the species possessing sequences from a single specimen, for which taxonomic revision is necessary. (DOC) [file pone.0021506.s007.doc]

**Table S5.** List of the species possessing sequences from a single specimen, for which taxonomic revision is necessary.

| Scientic Name | 窗体顶端  IInitial submitted name窗体底端 | Genbank accession No. | Reference |
| --- | --- | --- | --- |
| *Hynobius arisanensis* | *Hynobius formosanus* | Genome NC_008084 | [2] |
| *Hynobius guabangshanensis* | *Hynobius chinensis* | Genome NC_008088 | [2] |

The reference was given in Text S1.
