# Supplementary material for: Biogeographical Consequences of Cenozoic Tectonic Events within East Asian Margins: A Case Study of Hynobius Biogeography
Source: PLoS One. 2011 Jun 28;6(6):e21506. doi: 10.1371/journal.pone.0021506 (PMC3125272; doi:10.1371/journal.pone.0021506)
Supplement: Table S6 — Uncorrected p -distance between NC_008084 and the Cyt b fragments from identified specimens of the five Hynobius species in the Taiwan Island. (DOC) [file pone.0021506.s008.doc]

**Table S6.** Uncorrected *p*-distance between NC_008084 and the *Cyt b* fragments from identified specimens of the five *Hynobius* species in the Taiwan Island.

|  | NC_008084 | *H. arisansensis* | *H. formosansus* | *H. fuca* | *H. glacialis* | *H. sonani* |
| --- | --- | --- | --- | --- | --- | --- |
| *H. arisansensis* | 0.000-0.010 (0.003) | 0.001-0.014 (0.005) |  |  |  |  |
| *H. formosansus* | 0.066-0.073 (0.069) | 0.066-0.077 (0.056) | 0.001-0.037 (0.023) |  |  |  |
| *H. fuca* | 0.102-0.105 (0.103) | 0.099-0.106 (0.094) | 0.103-0.111 (0.090) | 0.001-0.023 (0.012) |  |  |
| *H. glacialis* | 0.054-0.054 (0.054) | 0.052-0.057 (0.053) | 0.059-0.065 (0.049) | 0.098-0.101 (0.093) | 0.000-0.000 (0.000) |  |
| *H. sonani* | 0.033-0.036 (0.035) | 0.031-0.040 (0.032) | 0.064-0.075 (0.057) | 0.099-0.108 (0.098) | 0.054-0.056 (0.054) | 0.000-0.006 (0.003) |

The range and averages (in parentheses) of uncorrected p-distance were given. For comparison, the intra- and inter-specific distances provided in the Table 2 of Lai and Lue [13] (this reference was given in Text S1) were also shown.
