# Supplementary material for: Biogeographical Consequences of Cenozoic Tectonic Events within East Asian Margins: A Case Study of Hynobius Biogeography
Source: PLoS One. 2011 Jun 28;6(6):e21506. doi: 10.1371/journal.pone.0021506 (PMC3125272; doi:10.1371/journal.pone.0021506)
Supplement: Text S1 — Origin of molecular data sources for Hynobius species. (DOC) [file pone.0021506.s009.doc]

**Text S1. Origin of molecular data sources for *Hynobius* species.**

In this study, all molecular data were obtained from GenBank. The majority of molecular data were provided by Macey et al. [1] and Zhang et al. [2] (Table S2). The dataset of Macey et al. [1] has been used in Larson et al. [3]. Other sources for our molecular data including Zhang et al. [4], Arnason et al. [5], Mueller et al. [6], Weisrock et al. [7], Frost et al. [8], Tominaga et al. [9], Matsui et al. [10], Oh et al. [11], Nishikawa et al. [12], Lai and Lue [13], Matsui et al. [14,15], Yoshikawa et al. [16], Zhang et al. [17], Sakamoto et al. [18], Okamoto et al. [19] and Zhang et al. [20] (Table S2). The molecular data covered ten mitochondrial loci, including *12S 16S*, *Cytb*, *ND2*, *tRNA-Val*, *tRNA-Trp*, *tRNA-Ala*, *tRNA-Asn*, *tRNA-Cys* and *tRNA-Tyr*. Among 44 selected taxa, 36 taxa (ca. 82%) possessed nine or all ten genes, while the rest eight taxa possessed one or three genes (Table S2).

There are thirty-two *Hynobius* species recognized so far [21]. The mitochondrial DNA sequences were available for thirty species (ca. 94%; Table S2). The scientific name of the sequences for five species, i.e. *H. arisanensis*, *H. guabangshanensis*, *H. yangi*, *H. yatsui* and *H. yiwuensis*, were revised from their original submitted names in Genbank according to recent taxonomy studies, as discussed below. For another species *H. chinensis*, though there are 18 sequences in Genbank, most of them were revised to be sequences of *H. guabangshanensis* and *H. yiwuensis*. There is only one valid *Cytb* sequence after taxonomic revision (EF076244; [20]). Based on personal communication with one of authors Prof. Wu, we confirmed that this sequence was amplified from a specimen sampled in the type locality of *H. chinensis*, Yichang, Hubei Province, China [21,22].

Except seven species (*Hynobius chinensis*, *H. formosanus*, *H. fuca*, *H. glacialis*, *H. katoi*, *H. maoershanensis* and *H. sonani*) with only *Cytb* gene, twenty-three *Hynobius* species possessed multiple or all ten genes (Table S2). For thirteen of the twenty-three species, sequences were from the same specimen, while for the rest ten species, sequences were from different sources and were concatenated together to represent the genetic characters of one species (Table S2). Below we show how we made the decision to combine these sequences for each species.

**Step 1. Sequences from a same specimen without taxonomic revision**

For each of eleven species listed in Table S4, the sequences were from a mitochondrial genome [2,11] or from the same specimen (i.e. with the same specimen number). Thus, we simply concatenated them to represent a certain species.

Specially, though from two sources [1,12] (see Table S2), the sequences of *Hynobius nigrescens* were amplified from the same specimen KUHE17924, sampled in Kami-machi, Miyagi Prefecture, Japan. Thus it was appropriate to combine these sequences.

**Step 2. Sequences from a same specimen with taxonomic revision**

Similar as the cases described above, the sequences of either species listed in Table S5 were amplified from the same specimen. However, the scientific name of the specimen was revised according to recent taxonomy studies (Table S5).

- *Hynobius arisansensis*

Lai and Lue [13] clarified species delimitation of five *Hynobius* species distributed in the Taiwan Island based on the *Cytb* fragments. The intra-specific genetic distances (uncorrected p-distance) ranged from 0.1% to 3.7%, while the inter-specific genetic distances ranged from 3.1% to 11.1% [13] (see also Table S6). In this study, the sequence for *H. arisansensis* (NC_008084; [2]) was initially submitted as *H. formosanus* in Genbank. To check its identification, we calculated the uncorrected p-distance between the *Cytb* fragment of NC_008084 and the sequences provided by Lai and Lue [13] in Mega 4. The result showed that the genetic distance between NC_008084 and *H.* *arisansensis* ranged from 0.0 % to 1.0%, which was in contrast to the much larger distances (3.3%-10.5%) between NC_008084 and other species including *H. formosanus* (Table S6). Thus, we considered that NC_008084 (*H. formosanus*) in Zhang et al. [2] should revised as *H. arisansensis*.

- *Hynobius guabangshanensis*

According to Frost [21], there are five *Hynobius* species distributed in Central China, namely, *H. amjiensis* and *H. yiwuensis* in Zhejiang Province, *H. chinensis* in Hubei and Fujian Province, *H. guabangshanensis* in Hunan Province, and *H. maoershanensis* in Guangxi Province. In this study, the sequence for *H. guabangshanensis* (NC_008088; [2]) was initially submitted as *H. chinensis* in Genbank. However, the specimen of NC_008088 was sampled from the type locality of *H. guabangshanensis*, Qiyang, Hunan Province, China [21,23]. Besides this geographical evidence, genetic analyses were also performed. As described above, there was only one valid *Cytb* sequence of *H. chinensis* (EF076244; [20]) in Genbank. A large genetic distance of 5.6% was found between NC_008088 and this *H. chinensis* sequence. However, the distances between NC_008088 and the sequences of *H. guabangshanensis* (*Cytb*: EF616473 and EF076245; *16S*: EF616474) were only ranging from 0.0% to 0.4%. Thus, we considered that NC_008088 (*H. chinensis*) in Zhang et al. [2] should be revised as *H. guabangshanensis.*

**Step 3. Sequences from different specimens but combined with direct evidence**

The sequences of the six species listed below were amplified from different specimens. However, based on geographic and genetic evidences, we could avoid the risk of potential cryptic species when concatenating the sequences from different specimens.

- *Hynobius boulengeri*

The sequences of *Hynobius boulengeri* came from three sources: Macey et al. [1], Tominaga et al. [9] and Matsui et al. [10] (Table S2). The *Cytb* gene (AB266675; [10]), the fragments of *12S* and *16S* (AB201671 and AB201706; [9]) were all amplified from the specimen KUHE25653. The sequences [AY915946 and AY915994] from Macey et al. [1], which covered the fragments of *12S* and *16S*, complete *ND2*, and six *tRNAs*, were amplified from another specimen KUHE25655. Both of the specimens were sampled from Kamikitayama-mura, Nara Prefecture, Japan [24]. Moreover, to avoid the potential risk of cryptic species, we calculated the uncorrected p-distance between AB201671 (KUHE25653) and AY915994 (KUHE25655), since there was an overlap of 598 bp between the two fragments of *12S*. The genetic distance turned out to be only 0.2%, thus we consider it appropriate to concatenate the sequences of the two specimens to represent the genetic characters of *H. boulengeri*.

- *Hynobius kimurae*

The sequences of *Hynobius kimurae* came from three sources: Macey et al. [1], Tominaga et al. [9] and Matsui et al. [10] (Table S2). The *Cytb* gene (AB266674; [10]) was amplified from speimen KUHE16689, sampled in Otsu-shi, Shiga Prefecture, Japan. The upstream fragment of *12S* (AB201670; [9]) and the midstream fragment of *16S* (AB201705; [9]) were amplified from an unnumbered specimen (KUHE-UN) sampled in Kyoto-shi, Kyoto Prefecture, Japan. The allozymic and morphological analyses indicated that the individuals of *H. kimurae* from the above two location grouped together (population 13 and 14 in Matsui et al. [25]; population 15 and 16 in Matsui et al. [26]). Thus it seemed proper to concatenate the sequences from specimens in the two locations. The midstream of *12S*, upstream of *16S*, complete *ND2*, and six *tRNAs* (AY915947 and AY915995; [1]) were amplified from another specimen KUHE22370 with the sampling site unknown. However, based on an overlap of *12S* fragments (592 bp), we calculated the uncorrected p-distance between AY915995 (KUHE22370) and AB201670 (KUHE-UN). Since the distance was only 0.2%, it was appropriate to concatenate these sequences when *H. kimurae* was represented.

- *Hynobius naevius*

The sequences of *Hynobius naevius* came from three sources: Macey et al. [1], Tominaga et al. [9] and Matsui et al. [10]. The The *Cytb* gene (AB266672; [10]), the fragments of *12S* and *16S* (AB201659 and AB201694; [9]) were all amplified from the specimen KUHE28584, sampled in Tara-cho, Saga Prefecture, Japan. The sequences [AY915937 and AY915985] from Macey et al. [1], which covered the fragments of *12S* and *16S*, complete *ND2*, and six *tRNAs*, were amplified from another specimen KUHE12984, sampled in Kitakyushu-shi, Fukuoka Prefecture, Japan. The two specimens were both identified as the Group A (large type) individuals of *Hynobius naevius* [9,27]. The Group A lineage of *H. naevius* was proved to be monphyletic [9] and was recognized as the true *H. naevius* [28]. Thus it was appropriate to combine these sequences.

- *Hynobius nebulosus*

The sequences of *Hynobius nebulosus* came from three sources: Macey et al. [1], Tominaga et al. [9] and Matsui et al. [14] (Table S2). The *Cytb* gene (AB445786; [14]), the fragments of *12S* and *16S* (AB201668 and AB201703; [9]) were all amplified from the same specimen KUHE24693. The sequences [AY915925 and AY915973] from Macey et al. [1], which covered the fragments of *12S* and *16S*, complete *ND2*, and six *tRNAs*, were amplified from another specimen KUHE24698. Both of the specimens were sampled from the same population in Isahaya-shi, Nagasaki Prefecture, Japan (population 4 in Matsui et al. [29]). Moreover, the uncorrected p-distance of the overlap *12S* fragments between AB201668 (KUHE24693) and AY915973 (KUHE24698) was only 0.2%. Thus, it was proper to concatenate these sequences.

- *Hynobius yangi*

*Hynobius yangi* was a recent reported species that has long been confused with *H. leechii* [30]. In our study, the sequences of *H. yangi* were combined from two different sources [1,13] with taxonomy revision from *H. leechii*. The fragments of *12S* and *16S*, complete *ND2*, and six *tRNAs* (AY915929 and AY915977; Macey et al. [1]) were amplified from *H. leechii* specimen HLc10 from South Korea. According to the Genbank submitted information, this specimen was marked with a note “Form C”. As indicated in Kim et al. [30], previous specimens of *H. leechii* Form C were now re-described as *H. yangi*. The *Cytb* gene (DQ652231; [13]) was amplified from *H. leechii* specimen NTNUB201703 from Pusan (= Busan-shi), South Korea, which was the type locality of *H. yangi* [30]. Thus, we also revised this specimen as *H. yangi*. Finally, we concatenated the sequences from the two specimens to represent the genetic characters of *H. yangi*.

- *Hynobius yatsui*

In a recent research, previous *Hynobius naevius yatsui* (smaller [B] of *H. naevius*) was reevaluated and elevated to full species rank as *H. yatsui* [31]. In this study, the sequences of *H. yatsui* were combined from Tominaga et al. [9] and Sakamoto et al. [18]. The *Cytb* gene (AB297522; [18]) was amplified from a *H. yatsui* specimen (see original literature), though it was submitted as *H. naevius* in Genbank. The *12S* and *16S* fragments (AB201663 and AB201698; [9]) was amplified from a ‘*H. naevius* (Type B)’ specimen KUHE24969, which was re-defined as *H. yatsui* in Tominaga and Matsui [31]. Both of the specimen were sampled in Saeki-shi (formerly Ume-machi), Oita Prefecture, Japan. Thus, these sequences were concatenated to represent the genetic characters of *H. yatsui*.

**Step 4. Sequences from different specimens but combined with indirect evidence**

The sequences of four species listed below were amplified from different specimens. However, both geographic and genetic evidences (i.e. direct evidences) were lacked to make the decision of combination. We concatenated the sequences with caution based on indirect evidences: e.g. the recognition of monophyly of the species in previous studies; no evidence for potential cryptic species that would affect our inference of phylogeny of *Hynobius*, etc.

Moreover, we split the sequences of four species into single individuals and built the phylogeny of *Hynobius* based on this new dataset. In other words, different specimens of the four species were treated as different analytic units in this tested phylogenetic inference. If the individuals of one species formed a monophyletic lineage, it would be reasonable for us to concatenate the sequences.

Based on indirect evidences and phylogenetic inference (Figure S1), we thought that error combinations could only happen for cryptic species forming a monophyletic lineage (if any), which would not result in a wrong phylogeny of *Hynobius*. Finally, because interested nodes in this study foucs on major clades, we considered the effect on our conclusions would be very little if any error combinations happened.

- *Hynobius lichenatus*

The sequences of *Hynobius lichenatus* came from two sources: Macey et al. [1] and Matsui et al. [10] (Table S2). The *Cytb* gene (AB266670; [10]) was amplified from specimen KUHE9404, sampled in Omagari-shi, Akita Prefecture, Japan. The fragments of *12S* and *16S*, complete *ND2*, and six *tRNAs* (AY915940 and AY915988; [1]) were amplified from another specimen J-9 with sampling site unknown. Direct genetic comparison of the sequences from different sources could not be performed as no overlap was found. As indirect evidence, Matsui et al. [10] recognized the monophyly of *H. lichenatus* based on *Cytb* and *D-loop* fragments. Moreover, after the review of previous literatures, we found no evidence about the possible cryptic species within *H. lichenatus* that would affect the resolution of the phylogeny of *Hynobius* species. Thus, we combined these sequences arbitrarily to represent the genetic characters of *H. lichenatus*.

- *Hynobius retardatus*

The sequences of *Hynobius retardatus* came from two sources: Macey et al. [1] and Matsui et al. [15] (Table S2). The *Cytb* gene (AB363609; [15]) were amplified from specimen KUHE13034, sampled in Ebetsu, Hokkaido, Japan. The fragments of *12S* and *16S*, complete *ND2*, and six *tRNAs* (AY915948 and AY915996; [1]) were amplified from specimen KUHE14545 with the exact sampling site unknown (undoubtedly it was in Hokkaido, Japan). We couldn’t calculate the uncorrected p-distance as no overlap was found between the sequences of the two specimens. However, the two specimens formed a monophyletic lineage on the phylogeny (Figure S1), thus we thought it reasonable to concatenate the sequences.

- *Hynobius tokyoensis*

The sequences of *Hynobius tokyoensis* came from two sources: Macey et al. [1] and Matsui et al. [10] (Table S2). The *Cytb* gene (AB266640; [10]) was amplified from specimen KUHE25836, sampled in Hachioji-shi, Tokyo Prefecture, Japan. The fragments of *12S* and *16S*, complete *ND2*, and six *tRNAs* (AY915941 and AY915989; [1]) were amplified from specimen KUHE16911 with the exact sampling site unknown. Matsui et al. [10] recognized the monophyly of *H. tokyoensis* based on a wide range of sampling using *Cytb* and *D-loop* fragments. Thus, we concatenated the sequences from different specimen though direct genetic comparison could not be performed.

- *Hynobius yiwuensis*

All the sequences of *Hynobius yiwuensis* were initially submitted as *H. chinensis* in Genbank. The *Cytb* sequence (DQ652229; [13]) was amplified from specimen NTNUB241795 sampled in Huantan, Zhejiang Province, China. The fragments of *12S* and *16S*, complete *ND2*, and six *tRNAs* (AY915934 and AY915982; [1]) were amplified from specimen TP24994 in Zhejiang Province, and were used as *H. yiwuensis* in Larson et al. [3]. According to Frost [21], *H. chinensis* was restricted to the populations in Hubei Province and Fujian Province. The populations in Zhejiang Province were now defined as *H. yiwuensis*, which was often considered as a synonym of *H. chinensis* [21]. Fu et al. [32] gave the molecular evidence for the distinctiveness and distribution range of *H. yiwuensis*. According to the geographic range defined in Fu et al. [32] and Frost [21], we revised these sequences as *H. yiwuensis*.

Fu et al. [32] described the genetic difference between the mainland and island populations within the monophyletic *H. yiwuensis*. We found that the *Cytb* sequence was from mainland population [13], but we could not make sure whether the sequences of Macey et al. [1] were from mainland or island population. However, the monophyly of *H. yiwuensis* was recognized in Fu et al. [32] The two specimen used in this study formed a monophyletic lineage on the phylogeny (Figure S1). Thus, the combination of the sequences seemed to be proper.

**Reference**

1. Macey JR, Weisrock DW, Fang Z, Matsui M, Larson A, et al. (submitted to Genbank in 2005) Molecular phylogenetics of the Hynobiidae (Amphibia: Caudata): evidence for an old northern Asian fauna.

2. Zhang P, Chen YQ, Zhou H, Liu YF, Wang XL, et al. (2006) Phylogeny, evolution, and biogeography of Asiatic salamanders (Hynobiidae). Proc Natl Acad Sci U S A 103: 7360–7365.

3. Larson A, Weisrock DW, Kozak KH (2003) Phylogenetic systematics of salamanders (Amphibia: Urodela), a review. In: Server DM, editor. Reproductive biology and phylogeny of Urodela (Amphibia). Enfield: NH Science Publishers. pp. 31-108.

4. Zhang P, Chen YQ, Liu YF, Zhou H, Qu LH (2003) The complete mitochondrial genome of the Chinese giant salamander, *Andrias davidianus* (Amphibia: Caudata). Gene 311: 93-98.

5. Arnason U, Gullberg A, Janke A, Joss J, Elmerot C (2004) Mitogenomic analyses of deep gnathostome divergence: a fish is a fish. Gene 333: 61-70.

6. Mueller RL, Macey JR, Jaekel M, Wake DB, Boore JL (2004) Morphological homoplasy, life history evolution, and historical biogeography of plethodontid salamanders inferred from complete mitochondrial genomes. Proc Natl Acad Sci U S A 101: 13820-13825.

7. Weisrock DW, Harmon LJ, Larson A (2005) Resolving deep phylogenetic relationships in salamanders: analyses of mitochondrial and nuclear genomic data. Syst Biol 54: 758-777.

8. Frost DR, Grant T, Faivovich J, Bain RH, Haas A, et al. (2006) The amphibian tree of life. Bull Am Mus Nat Hist 297: 1-291.

9. Tominaga A, Matsui M, Nishikawa K, Tanabe S (2006) Phylogenetic relationships of *Hynobius naevius* (Amphibia: Caudata) as revealed by mitochondrial 12S and 16S rRNA genes. Mol Phylogenet Evol 38: 677-684.

10. Matsui M, Tominaga A, Hayashi T, Misawa Y, Tanabe S (2007) Phylogenetic relationships and phylogeography of *Hynobius tokyoensis* (Amphibia: Caudata) using complete sequences of cytochrome b and control region genes of mitochondrial DNA. Mol Phylogenet Evol 44: 204-216.

11. Oh DJ, Chang MH, Oh HS, Jung YH (2007) The complete mitochondrial DNA sequence of the Jeju salamander, *Hynobius quelpaertensis*, and the phylogenetic relationships among the Hynobiidae. Korean J Genet 29: 331-341.

12. Nishikawa K, Jiang JP, Matsui M, Mo YM, Chen XH, et al. (2010) Invalidity of *Hynobius yunanicus* and molecular phylogeny of *Hynobius* salamander from continental China (Urodela, Hynobiidae). Zootaxa 2426: 65-67.

13. Lai JS, Lue KY (2008) Two new *Hynobius* (Caudata: Hynobiidae) salamanders from Taiwan. Herpetologica 64: 63-80.

14. Matsui M, Tominaga A, Liu WZ, Tanaka-Ueno T (2008) Reduced genetic variation in the Japanese giant salamander, *Andrias japonicas* (Amphibia: Caudata). Mol Phylogenet Evol 49: 318-326.

15. Matsui M, Yoshikawa N, Tominaga A, Sato T, Takenaka S, et al. (2008) Phylogenetic relationships of two *Salamandrella* species as revealed by mitochondrial DNA and allozyme variation (Amphibia: Caudata: Hynobiidae). Mol Phylogenet Evol 48: 84-93.

16. Yoshikawa N, Matsui M, Nishikawa K, Kim JB, Kryukov A (2008) Phylogenetic relationships and biogeography of the Japanese clawed salamander, *Onychodactylus japonicas* (Amphibia: Caudata: Hynobiidae), and its congener inferred from the mitochondrial cytochrome b gene. Mol Phylogenet Evol 49: 249-259.

17. Zhang P, Papenfuss TJ, Wake MH, Qu LH, Wake DB (2008) Phylogeny and biogeography of the family Salamandridea (Amphibia: Caudata) inferred from complete mitochondrial genomes. Mol Phylogenet Evol 49: 586-597.

18. Sakamoto M, Tominaga A, Matsui M (2009) Phylogeography of *Hynobius yatsui* (Amphibia: Caudata) in Kyushu, Japan. Zool Sci 26: 35-47.

19. Okamoto K, Suizu Y, Mizuno K, Tanigawa S, Sako K, et al. (unpublished) The giant salamander - comparison of mitochondrial whole genome between the Japanese and Chinese species.

20. Zhang Y, Wu M, Wang WJ, Wang X (unpublished) Direct submission to Genbank. (Note: EF076244 from a specimen of the type location for *Hynobius chinensis*. Personal communications with one of authors, Prof. Wu M.)

21. Frost DR (2009) Amphibian Species of the World: an Online Reference. Version 5.3 (12 February, 2009). Electronic Database accessible at http://research.amnh.org/herpetology/amphibia/. New York: American Museum of Natural History.

22. Wang X, Wu M, Zhang Y, Wang WJ, Liu MY, et al. (2007) On the re-discovery of *Hynobius chinensis* Günther, 1989 from type-locality and its description after 116 years. Sichuan J Zool 26: 57-58.

23. Shen YH, Deng XJ, Wang B (2004) A new hynobiid species *Hynobius guabangshanensis* from Hunan Province, China (Amphibia: Hynobiidae). Acta Zool Sinica 50: 209-215.

24. Nishikawa K, Matsui M, Tanabe S (2005) Biochemical phylogenetics and historical biogeography of *Hynobius boulengeri* and *H. stejnegeri* (Amphibia: Caudata) from the Kyushu region, Japan. Herpetologica 61: 54-62.

25. Matsui M, Misawa Y, Nishikawa K, Tanabe S (2000) Allozymic variation of *Hynobius kimurae* Dunn (Amphibia, Caudata). Comp Biochem Physiol B 125: 115-125.

26. Matsui M, Misawa Y, Nishikawa K (2009) Morphological variation in a Japanese salamander, *Hynobius kimurae* (Amphibia, Caudata). Zool Sci 26: 87-95.

27. Tominaga A, Matsui M, Nishikawa K, Sato S (2003) Occurrence of two types of *Hynobius naevius* in Northern Kyushu, Japan (Amphibia: Urodela). Zool Sci 20: 1467-1476.

28. Tominaga A, Matsui M (2007) Estimation of the type locality of *Hynobius naevius* (Temminck and Schlegel, 1838), a salamander from Japan (Amphibia: Caudata). Zool Sci 24: 940-944.

29. Matsui M, Nishikawa K, Utsunomiya T, Tanabe S (2006) Geographic allozyme variation in the Japanese clouded salamander, *Hynobius nebulosus* (Amphibia: Urodela). Biol J Linn Soc Lond, 89: 311-330.

30. Kim JB, Min MS, Matsui M (2003) A new species of lentic breeding Korean salamander of the genus *Hynobius* (Amphibia: Urodela). Zool Sci 20: 1163-1169.

31. Tominaga A, Matsui M (2008) Taxonomic status of a salamander species allied to *Hynobius naevius* and a reevaluation of *Hynobius naevius yatsui* Oyama, 1947 (Amphibia, Caudata). Zool Sci 25: 107-114.

32. Fu JZ, Hayes M, Liu ZJ, Zeng XM (2003) Genetic divergence of the southeastern Chinese salamanders of the genus *Hynobius*. Acta Zool Sinica 49: 585-591.
