# Supplementary material for: Biogeographical Consequences of Cenozoic Tectonic Events within East Asian Margins: A Case Study of Hynobius Biogeography
Source: PLoS One. 2011 Jun 28;6(6):e21506. doi: 10.1371/journal.pone.0021506 (PMC3125272; doi:10.1371/journal.pone.0021506)
Supplement: Text S2 — Re-examination of relationships among Hynobius Clades 1–5 defined in Figure 3 using reduced taxon dataset. (DOC) [file pone.0021506.s010.doc]

**Text S2. Phylogenetic analyses on *Hynobius* in Clade 1-5.**

**Methods**

In the partitioned Bayesian (BA) and maximum likelihood (ML) analyses, *Hynobius* salamanders were divided into nine major clades with strong supports for most nodes (Figure 3). However, the monphyly of Clade 2 (BS = 36%; PP = 0.79) and the sister-group relationship of Clade 1 and Clade 2 (BS = 58%; PP = 0.82) were inferred with relatively poor supports. In order to resolve the above relationships, we performed a further analysis on the *Hynobius* species in Clades 1-5. The four species possessed only one or three genes, i.e. *H. chinensis*, *H. maoershanensis*, *H. katoi*, *H. yatsui* (see Table S2), were excluded in the analysis. The five species in Clade 6 were used as the outgroup taxa.

For the partitioned BA analysis, independent substitution models were first selected for each partition using the Akaike information criterion (AIC) implemented in jModeltest ver. 0.1.1 [1]. Markov chains Monte Carlo (MCMC) were run for 10 million generations with a sampling frequency of 1000 in MrBayes ver. 3.1.2 [2]. Stationarity was checked in Tracer ver. 1.4.1 [3]. Then, the first one million generations before stationarity were discarded as burnin and the remaining trees were used to build a consensus tree.

Partitioned maximum likelihood (ML) analysis was implemented using a rapid-hill-climbing algorithm in RAxML ver. 7.0.4 [4]. First, the best-scoring ML tree was inferred with 100 replications under the GTRGAMMA model. Then, a nonparametric bootstrap analysis of 1000 replications was conducted under the GTRGAMMA model to evaluate node robustness of the ML tree. The GTACAT model was not applied because of the small dataset with only 18 taxa.

**Results**

Using a reduced taxon sampling, the phylogenetic relationships among the Clades 1-5 were revealed to be accordant with the result from the full dataset (Figure 3 and Figure S2). The monophyly of Clade 2 was inferred with strong supports (BS = 87%; PP = 1.00), but the sister-group relationship of Clade 1 and Clade 2 was still inferred with moderate support (BS = 57%; PP = 0.83).

**Reference**

1. Posada D (2008) jModeltest: phylogenetic model averaging. Mol Biol Evol 25: 1253-1256.

2. Huelsenbeck JP, Ronquist F (2001) Mrbayes: Bayesian inference of phylogenetic trees. Bioinformatics 17: 754-755.

3. Rambaut A, Drummond AJ (2007) Tracer v 1.4. Available from http://beast.bio.ed.ac.uk/Tracer.

4. Stamatakis A (2006) RAxML-VI-HPC: maximum likelihood-based phylogenetic analyses with thousands of taxa and mixed models. Bioinformatics 22: 2688–2690.
